# Efficient synthesis of 3-alkyl-2-(-1*H*-1,2,3-triazolyl)methyl)thio)-2,3-dihydroquinazolin-4(1*H*)-one derivative via multistep synthesis approach by novel Cu@Py-Oxa@SPION catalyst

**DOI:** 10.1186/s13065-023-01072-4

**Published:** 2023-11-14

**Authors:** Alireza Sherafati, Shahram Moradi, Mohammad Mahdavi

**Affiliations:** 1 Department of Chemistry Tehran North Branch, Islamic Azad University, Tehran, Iran; 2 Endocrinology and Metabolism Research Centre, Endocrinology and Metabolism Clinical Sciences Institute, Tehran University of Medical Sciences, Tehran, Iran

**Keywords:** Copper catalyst, SPION, Click reaction, 1,2,3-Triazolylthio-2,3-dihydroquinazolinone, Triazole, Quinazolinone

## Abstract

In this pared, an efficient method is introduced for the synthesis of 3-alkyl-2-(((4-(2-oxopropyl)-1*H*-1,2,3-triazol-1-yl)alkyl)thio)-2,3-dihydroquinazolin-4(1*H*)-one derivatives. These novel products have both 1,2,3-triazole and quinazolinone in their structures. For the synthesis of these products, a novel catalyst is designed, synthesized, and characterized by the immobilization of copper onto modified magnetic iron oxide. The catalyst (denoted: Cu@Py-Oxa@SPION) was characterized by several characterization techniques. In this regard, 16 3-alkyl-2-(((4-(2-oxopropyl)-1*H*-1,2,3-triazol-1-yl)alkyl)thio)-2,3-dihydroquinazolin-4(1*H*)-one derivatives were synthesized in high isolated yields (77–86%). As an advantage, the catalyst is highly recoverable and its activity has not decreased after 7 sequential runs. The method is very efficient for the synthesis of the products in high isolated yields under mild reaction conditions in a green solvent. The scope of the method is broad and several examples were successfully synthesized using starting materials with different functional groups.

**Supplementary Information:**

The online version contains supplementary material available at 10.1186/s13065-023-01072-4.

## Introduction

Catalytic reactions are of high interest, due to their advantages in organic synthesis, including high isolated yields of the products, milder reaction conditions, and performing the reactions in green and environmentally friendly solvents. These advantages have led to several studies on developing catalysts to overcome their drawbacks, including difficulties in separation from the reaction mixture and its reusability. An interesting approach for this purpose is to immobilize the catalysts onto nanoparticles [1–9]. Among different nanoparticles, magnetic nanoparticles in general, and iron oxide nanoparticles in particular, are interesting nanoparticles to be used as support for the catalysts. Iron oxide nanoparticles are chemically and physically stable, magnetically separable, and easily synthesized and functionalized. Therefore, these nanomaterials are of high interest to be used as support for catalysts [10–18]. Copper is a transition metal that is used as a catalyst for several chemical transformations [19–21]. This metal has been used as a catalyst in several reactions, including oxidation [22–24], reduction [25, 26], carbon–carbon coupling [27–29], and alkyne-azide cycloaddition click reaction [30–33].

**Scheme 1:**
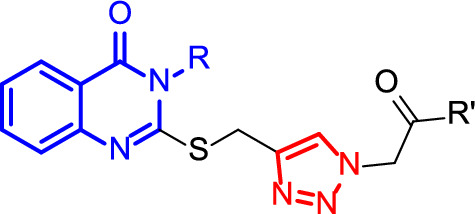
Chemical structure of 3-alkyl-2-(((4-(2-oxopropyl)-1*H*-1,2,3-triazol-1-yl)alkyl)thio)-2,3-dihydroquinazolin-4(1*H*)-one containing quinazilinone and 1,2,3-triazole

Copper(I)-catalyzed alkyne-azide cycloaddition click reaction is an interesting and efficient reaction for the synthesis of 1,2,3-triazole derivatives from the reaction of an alkyne and an alkyl azide. This reaction is fast and efficient and the products are easily synthesized in high isolated yields using copper(I) as the catalyst [34–36]. 1,2,3-Triazolea are significant due to their valuable properties as antiplatelet [37], dye [38], antioxidant [39, 40], antimicrobial [41–43], and anticancer [44–46]. On the other hand, quinazolinone and its derivatives have shown vast biological properties, such as anticonvulsant [47], anticancer [48–50], and antibacterial [51–53] properties. In addition, quinazolinone derivatives have enhanced the lubrication properties of oil [54–56]. Therefore, several efforts have been focused on the synthesis of the quinazolinones, especially using green solvent and conditions [57–63]. Regarding the significance of quinazilinones and 1,2,3-triazoles in modern medicinal chemistry, in this paper, we report the synthesis of novel 3-alkyl-2-(((4-(2-oxopropyl)-1*H*-1,2,3-triazol-1-yl)alkyl)thio)-2,3-dihydroquinazolin-4(1*H*)-one derivative (Scheme 1).

The synthesis is performed in 4 steps with commercially available chemicals in efficient and facile methods. The synthesis of the products is presented in Scheme 4. Novel Cu@Py-Oxa@SPION catalyst is introduced and synthesized for alkyne-azide cycloaddition click reaction in this synthesis.

## Results and discussion

**Scheme 2:**
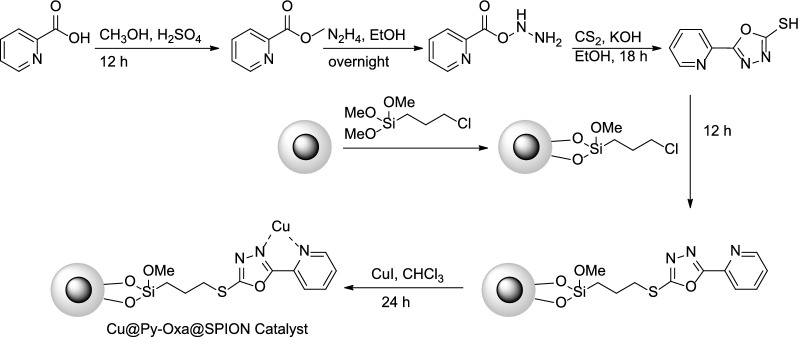
Synthesis of Cu@Py-Oxa@SPION catalyst

In this study, we have introduced a highly efficient and eco-friendly method for the multistep synthesis of 3-alkyl-2-(((4-(2-oxopropyl)-1*H*-1,2,3-triazol-1-yl)alkyl)thio)-2,3-dihydroquinazolin-4(1*H*)-one derivatives. These novel products possess unique structures that combine the 1,2,3-triazole and quinazolinone moieties, making them valuable compounds in the field of medicinal chemistry. The key feature of this study is the development of a novel catalyst, denoted as Cu@Py-Oxa@SPION, which is based on the immobilization of copper onto superparamagnetic iron oxide nanoparticles (SPION). This catalyst was synthesized via a simple and straightforward process that involved the encapsulation of SPION by silica, followed by functionalization with 2-(pyridin-2-yl)-1,3,4-oxadiazole, which was used as a ligand for the copper catalyst. The entire synthesis process of the catalyst is presented in detail in Scheme 2. The newly developed catalyst showed remarkable performance in the synthesis of the target compounds, yielding high isolated yields (77–86%) under mild reaction conditions and in a green solvent. Importantly, the catalyst demonstrated excellent recyclability, with no loss of activity observed after 7 sequential runs. This characteristic is particularly significant, as it not only enhances the sustainability of the process but also makes it economically viable.

**Fig. 1:**
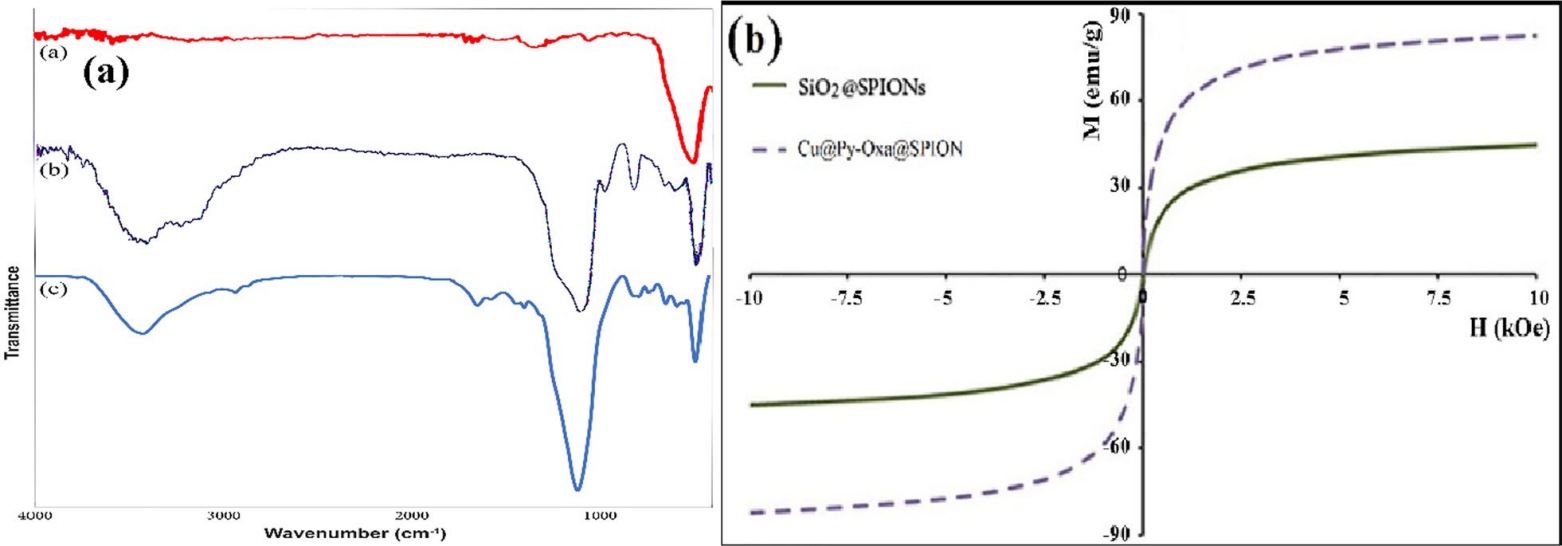
**a** FTIR; **b** VSM results of Cu@Py-Oxa@SPION catalyst. In the FTIR spectra, the red line (denoted a) shows SiO_2_ encapsulated SPION the black line (denoted b) shows SiO_2_ encapsulated SPION and the blue line (denoted c) shows Cu@Py-Oxa@SPION catalyst

After the synthesis of the catalyst, Cu@Py-Oxa@SPION was fully characterized by several characterization techniques. The successful synthesis of the catalyst was studied by FT-IR spectroscopy. The FT-IR spectrum of Cu@Py-Oxa@SPION is presented in Fig. 1a. Figure 1a represents silica coated SPION. The characteristic peaks at 1078 and 583 cm^−1^ belong to Si–O and Fe–O vibrations, respectively. Hydroxyl group vibration of the catalyst could be observed at 3328 cm^−1^. The magnetic properties of Cu@Py-Oxa@SPION catalyst are presented in Fig. 1b. for better comparison, the VSM results of silica coated SPION and Cu@Py-Oxa@SPION catalyst are compared in Fig. 1b. It is clearly observed that the catalyst shows superparamagnetic behavior. A decrease in the magnetization of the catalyst could be correlated to the functionalization of the magnetic nanoparticles by different groups. However, the magnetization is intense enough for the magnetical separation of Cu@Py-Oxa@SPION catalyst from the reaction mixture. For the determination of the copper content in the structure of the catalyst, ICP analysis was used. The results showed that each gram of Cu@Py-Oxa@SPION catalyst contains 3.71 mmol of copper (Additional file 1).

**Fig. 2:**
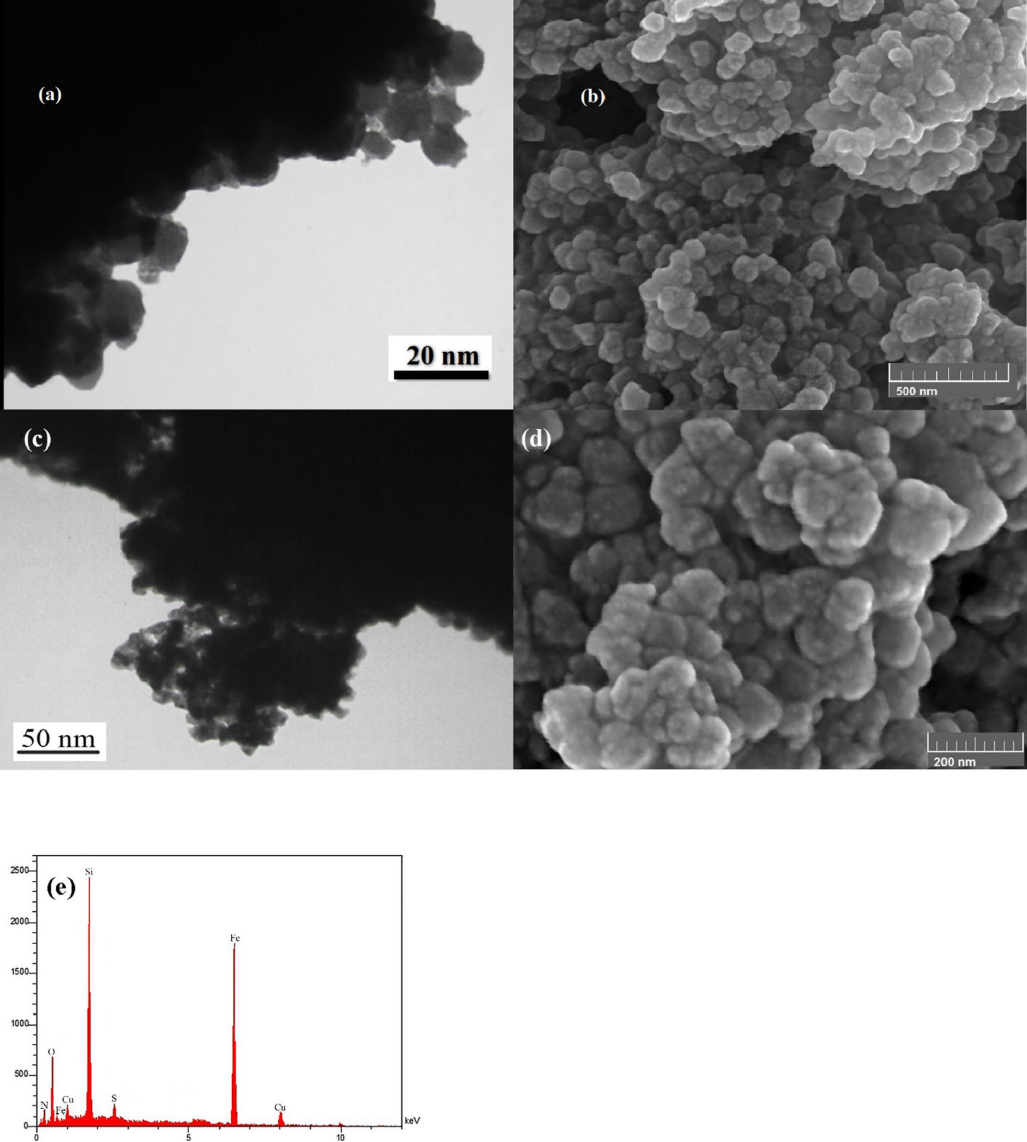
**a**, **c** SEM; and **b**, **d** TEM images; and **e** EDS result of Cu@Py-Oxa@SPION catalyst with different magnifications

The structures of Cu@Py-Oxa@SPION catalyst were studied by SEM and TEM microscopy methods. The SEM and TEM images are presented in Fig. 2a, d. Based on the results, the nanoparticles are spherical with an average size of 22 nm with a very narrow polydispersity. The particles are uniform and no aggregation or agglomeration was observed, which could due to the functionalization of SPION by silica and 2-(pyridin-2-yl)-1,3,4-oxadiazoleis, which prevents the aggregation of the nanoparticles. In addition, energy-dispersive X-ray spectroscopy (EDS) analysis was carried out to confirm the presence of copper in the catalyst (Fig. 2e). The EDS spectrum obtained showed the characteristic peaks of copper at the appropriate energy level, indicating the successful incorporation of copper into the catalyst structure. The intensity of the copper peak was strong, suggesting a high percentage of copper in the catalyst. The EDS analysis provided clear evidence of the presence of copper in the catalyst and supported the characterization data obtained from other techniques. Overall, the EDS analysis confirmed the successful synthesis of the Cu@Py-Oxa@SPION catalyst and its potential for use in catalytic reactions.

After the characterization of Cu@Py-Oxa@SPION, the catalyst was used for the synthesis of 3-alkyl-2-(((4-(2-oxopropyl)-1*H*-1,2,3-triazol-1-yl)alkyl)thio)-2,3-dihydroquinazolin-4(1*H*)-ones. As could be seen in Scheme 2, the challenging step of the reaction is the last one, which involves the click reaction for the formation of 1,2,3-triazole heterocycle. Therefore, this reaction was optimized by performing the reaction in different solvents, in the presence of various amounts of Cu@Py-Oxa@SPION. In addition, the reaction is performed in the presence of non-immobilized CuI to compare the results of the synthesis of the desired products by Cu@Py-Oxa@SPION and nonimmobilized copper catalyst. The results are presented in Table 1. It could be observed that Cu@Py-Oxa@SPION has acted much more efficiently than CuI. In addition, the presence of Cu@Py-Oxa@SPION catalyst is essential for the reaction performance, while performing the reaction without the catalyst has not led to the product.

**Table 1:** Optimization of the reaction conditions

Entry	Catalyst	Amount (mg)	Solvent	Isolated yield (%)
1	No catalyst	0	EtOH	0
2	Cu@Py-Oxa@SPION	20	EtOH	17
3	Cu@Py-Oxa@SPION	40	EtOH	50
4	Cu@Py-Oxa@SPION	50	EtOH	86
5	Cu@Py-Oxa@SPION	70	EtOH	86
6	Cu@Py-Oxa@SPION	50	H_2_O	0
7	Cu@Py-Oxa@SPION	50	CH_2_Cl_2_	36
8	Cu@Py-Oxa@SPION	50	DMF	24
9	Cu@Py-Oxa@SPION	50	MeOH	62
10	Py-Oxa@SPION	50	EtOH	0
11	CuI	50	EtOH	51
12	Cu@Py-Oxa@SPION^a^	50	EtOH	86

Reaction conditions: 3-benzyl-2-(prop-2-yn-1-ylthio)quinazolin-4(3*H*)-one (1 mmol), *N*-benzyl-2-chloroacetamide (1 mmol), NaN_3_ (1.2 mmol),solvent (5 mL), r.t, 24 h

^a^The reaction was performed under reflux conditions

Having the optimized reaction conditions, the scope and the generality of the method was evaluated by using different substrates. The results are presented in Table 2. It should be observed that all the substrates have given the corresponding products in high isolated yields.

**Table 2:** Scope and generality of Cu@Py-Oxa@SPION catalyzed synthesis of 3-alkyl-2-(-1*H*-1,2,3-triazolyl)methyl)thio)-2,3-dihydroquinazolin-4(1*H*)-one derivative


Entry	R	R′	Yield (%)
1	*i*-Pr	2,4-Dimethyl phenyl	81
2	*i*-Pr	3-Chloro phenyl	79
3	*i*-Pr	4-Bromo phenyl	85
4	*i*-Pr	4-Methyl phenyl	83
5	*i*-Pr	4-Nitro phenyl	80
6	*i*-Pr	Phenyl	82
7	Phenyl	3,5-Dimethyl phenyl	77
8	Phenyl	3-Chloro phenyl	80
9	Phenyl	4-Bromo phenyl	81
10	Phenyl	4-Nitro phenyl	84
11	Phenyl	Phenyl	86
12	Benzyl	3,5-Dimethyl phenyl	85
13	Benzyl	4-Bromo phenyl	82
14	Benzyl	4-Nitro phenyl	84
15	Benzyl	Phenyl	85
16	Benzyl	3-Chloro phenyl	78

Reaction conditions: 3-benzyl-2-(prop-2-yn-1-ylthio)quinazolin-4(3*H*)-one (3 mmol), *N*-benzyl-2-chloroacetamide (3 mmol), NaN_3_ (3.6 mmol),solvent (10 mL), Cu@Py-Oxa@SPION catalyst (50 mg), r.t, 24 h

**Fig. 3:**
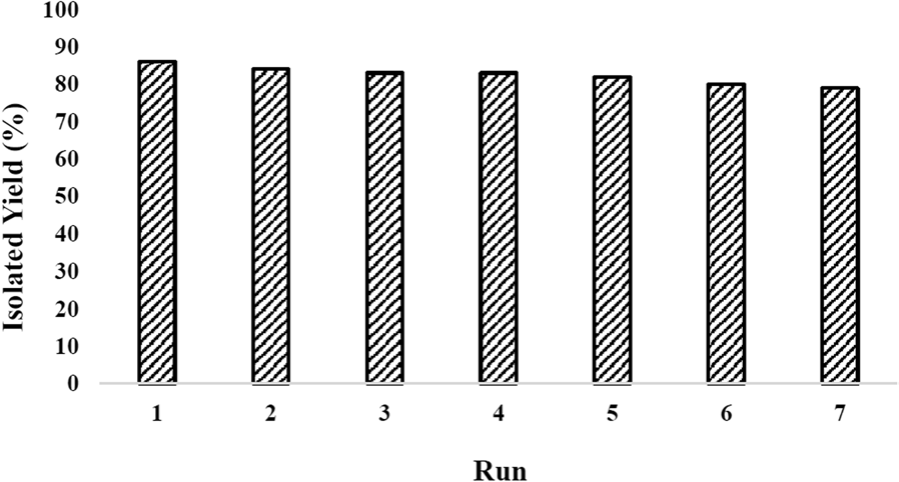
The recovery of Cu@Py-Oxa@SPION catalyst

As an advantage, Cu@Py-Oxa@SPION catalyst is its reusability. For studying the reusability of the catalyst, Cu@Py-Oxa@SPION was separated from the reaction mixture and used in the next reaction without any further purification. The recovery of the catalyst was repeated for 7 sequential reactions. The results are presented in Fig. 3. It could be observed that the activity of the catalyst has remained constant after various reaction runs. In addition, for studying the leaching of the catalyst, a model reaction was performed and the reaction mixture was stirred for 12 h. After 12 h, the catalyst was separated by an external magnet and the solution was analyzed for the presence of copper. During the recyclability studies, the stability of the Cu@Py-Oxa@SPION catalyst was evaluated by analyzing the copper content in the reaction solution using Inductively Coupled Plasma (ICP) analysis. The results showed that there was no detectable copper in the solution, indicating the high stability and reusability of the catalyst. In addition, in another reaction, the catalyst was separated from the reaction mixture before the completion of the reaction. The performance of the reaction was studied after the removal of the catalyst by GC and the results showed no performance in the reaction that confirms the necessity of the presence of the catalyst for the reaction.

**Fig. 4:**
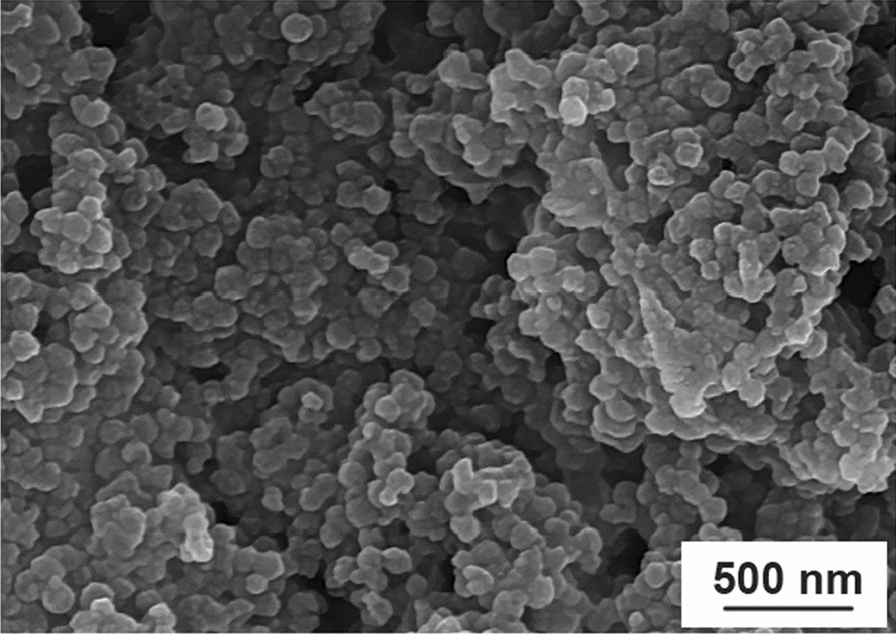
SEM image of Cu@Py-Oxa@SPION catalyst

For the best characterization of the stability of the catalyst, Cu@Py-Oxa@SPION catalyst was characterized by SEM. The SEM of the recovered catalyst is presented in Fig. 4. Based on the result, the catalyst did not show change in its structure after being used in the reaction.

In comparison to previously reported catalysts for the synthesis of 1,2,3-triazole-containing compounds, the Cu@Py-Oxa@SPION catalyst showed excellent performance. For instance, the Cu@Py-Oxa@SPION catalyst had a shorter reaction time and a lower catalyst loading compared to previously reported catalysts. Furthermore, the catalyst exhibited high stability and reusability, retaining its activity after multiple runs. In addition, the methodology presented in this work is a green and efficient synthesis route using a benign solvent, which makes it more environmentally friendly compared to other reported methods.

**Scheme 3:**
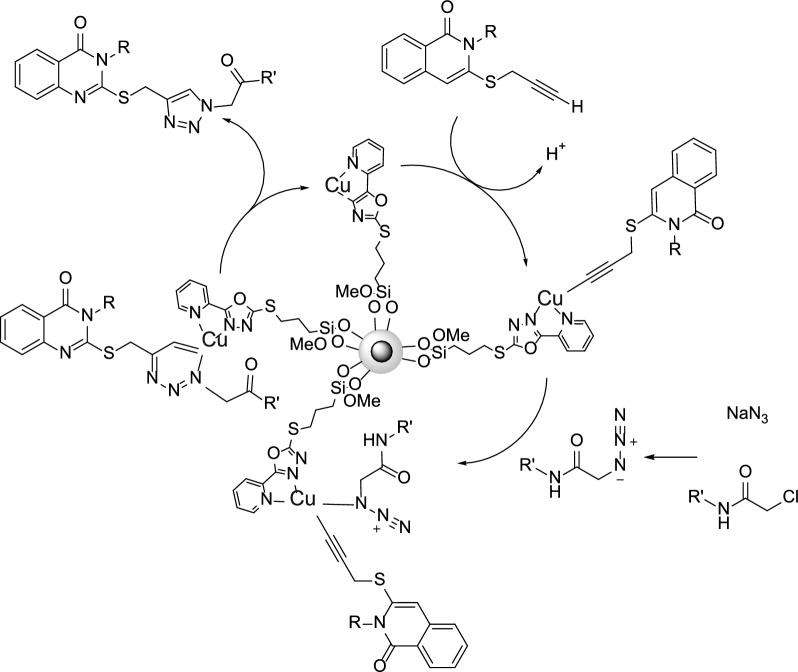
Possible mechanism for the synthesis of 3-alkyl-2-(((4-(2-oxopropyl)-1*H*-1,2,3-triazol-1-yl)alkyl)thio)-2,3-dihydroquinazolin-4(1*H*)-one products using Cu@Py-Oxa@SPION catalyst

A possible mechanism was proposed and suggested for the synthesis of 3-alkyl-2-(((4-(2-oxopropyl)-1*H*-1,2,3-triazol-1-yl)alkyl)thio)-2,3-dihydroquinazolin-4(1*H*)-one products using Cu@Py-Oxa@SPION catalyst. The suggested mechanism is presented in Scheme 3. According to the suggested mechanism, the presence of the catalyst is critical for the synthesis of the product. The copper attached to the catalyst play an important role in the activation of the alkyne, which subsequently undergoes nucleophilic attack by the azide group. The resulting intermediate then undergoes cyclization with the help of the copper catalyst to form the desired triazole ring. The immobilization of the copper onto the SPION nanoparticles is believed to enhance the stability and reusability of the catalyst, making it an attractive and efficient option for this type of multistep synthesis.

## Experimental

### General remarks

Solvents, reagents, and chemicals were obtained from Merck (Germany) and Fluka (Switzerland) Chemical Companies. Melting points were taken on a Kofler hot stage apparatus and are uncorrected. The IR spectra were obtained on a Nicolet Magna FT-IR 550 spectrophotometer (potassium bromide disks). Nuclear magnetic resonance spectra were recorded on Bruker FT-500 spectrometers using tetramethyl silane (TMS) as the internal standard in pure deuterated solvents. Chemical shifts are given in the δ scale in parts per million (ppm) and singlet (s), doublet (d), triplet (t), multiplet (m), and doublets of doublet (dd) are recorded. Mass spectra were recorded on an Agilent Technology (HP) mass spectrometer operating at an ionization potential of 70 eV. The elemental analysis was performed with an Elemetar Analysen system GmbH VarioEL CHNS mode. Purification of all products was conducted by column chromatography on silica gel using petroleum ether and ethyl acetate as eluent. Thin layer chromatography was carried out on silica gel 254 analytical sheets obtained from Fluka. Column chromatography was carried out on the column of silica gel 60 Merck (230–240 mesh) in glass columns (2 or 3 cm diameter) using 15–30 g of silica gel per one gram of the crude mixture. Transition electron microscope images were recorded on a HITACHI S-4160.

### Synthesis of silica encapsulated SPION

For the preparation of SPION, two micro-emulsions were prepared. The first one contained a solution of FeCl_3_·6H_2_O (202 mg), FeCl_2_·4H_2_O (75 mg), and cetyltrimethylammonium bromide (CTAB) (1.8 g) in 2.045 mL of deionized water as the aqueous phase, and toluene (29 mL) as the organic phase. The second micro-emulsion contained 1.8 g of CTAB in 29 mL toluene and 25% ammonium hydroxide solution (2.65 mL). The two micro-emulsions were mixed in a three-necked flask under vigorous stirring at 7000 rpm at 50 °C under a constant flow of N_2_ gas for 60 min. The dark precipitates were separated using an external magnet and washed with boiling EtOH from impurities. The obtained SPIONs were dried under vacuum for 12 h at room temperature.

For coating magnetic nanoparticles with silica, a solution of 200 µL of tetraethyl orthosilicate (TEOS) in 40 mL ethanol was added to a mixture containing 1 mg of SPION in 10 mL of deionized water under vigorous mechanical stirring followed by the addition of 1.25 mL of aqueous ammonia (25% w/w). After 1 h, the obtained magnetic nanoparticles suspension was magnetically separated and washed twice with deionized water. The SiO_2_@SPION product was separated and dried under reduced pressure at room temperature for 12 h.

### Synthesis of 2-(pyridin-2-yl)-1,3,4-oxadiazoleis ligand

Picolinic acid (10 mmol, 1.231 g) was weighed and added to methanol (15 mL). Then, sulfuric acid (100 µL) was added to the solution and stirred at room temperature for 12 h. the reaction mixture was neutralized by the addition of a 0.1 M solution of NaOH. The solvent was evaporated and the product was recrystallized from ethanol. In the next step, methyl picolinate product (5 mmol, 0.685 g) was dissolved in ethanol, and hydrazine (7 mmol, 0.2240 mg) was added. The reaction was stirred at room temperature overnight and the product was purified after the removal of the catalyst by recrystallization from ethyl acetate to form pure 2-((hydrazinyloxy)carbonyl)pyridine. In the next step, 2-((hydrazinyloxy)carbonyl)pyridine (4 mmol, 0.612 g) and KOH (5 mmol, 0.280 g) dissolved in ethanol (15 mL) and then CS_2_ (4 mmol, 0.304 g) was added. The reaction mixture was stirred under reflux conditions for 18 h. After the reaction was completed, 5-(pyridin-2-yl)-1,3,4-oxadiazole-2-thiol ligand was purified by recrystallization from ethanol.

### Synthesis of Cu@Py-Oxa@SPION catalyst

Silica encapsulated SPION (500 mg) was added to dry ethanol (10 mL) and sonicated for 30 min. after that, 3-(chloropropyl)-trimethoxysilane (10 mmol, 1.987 g) was added and stirred at 70 °C for 12 h. after that, the product was separated from the reaction mixture by a magnet and washed 3 times by toluene, ethanol, and acetone, respectively. The product was added to dry toluene (10 mL) and sonicated for 30 min. Then, 5-(pyridin-2-yl)-1,3,4-oxadiazole-2-thiol ligand (3 mmol, 0.540 g) was added and stirred for 24 h at room temperature. Then, 75 mL of CuCl (0.04 M) was added to a mixture of the above product in dichloromethane and was allowed to stir at r. t. overnight. The product was separated, washed with water, and EtOH, and then dried at r. t. The Cu@Py-Oxa@SPION nanocatalyst was obtained as a dark powder. After that, Cu@Py-Oxa@SPION catalyst was separated and washed with toluene, ethanol, and acetone and dried in a vacuum oven for 24 h.

### Synthesis of 3-alkyl-2-(((4-(2-oxopropyl)-1*H*-1,2,3-triazol-1-yl)alkyl)thio)-2,3-dihydroquinazolin-4(1*H*)-one derivatives

In the first step, isatoic anhydride (10 mmol, 1.630 g) was added to water, and amine (12.5 mmol) was added. The reaction mixture was stirred at room temperature and the reaction performance was monitored by TLC (hexane/ethyl acetate, 5:95). After the completion of the reaction, the product was filtered and washed with cold water, and dried in air. To the product (5 mmol) in ethanol (10 mL), was added CS_2_ (5 mmol, 0.380 g) and stirred under reflux conditions. After the reaction was completed, it was poured onto ice water and the product was filtered and purified by recrystallization from ethyl acetate. To this 3-alkyl-2-thioxo-2,3-dihydroquinazolin-4(1*H*)-one product (5 mmol) in DMF (20 mL), was added K_2_CO_3_ (8 mmol, 1.104 g) and propargyl bromide (5 mmol, 0,585 g) and stirred at 75 °C for 12 h. After the reaction was completed, the 3-alkyl-2-(prop-2-yn-1-ylthio)quinazolin-4(3*H*)-one product was separated by the addition of the reaction mixture to the ice water mixture, followed by washing in cold water 3 times.

**Scheme 4:**
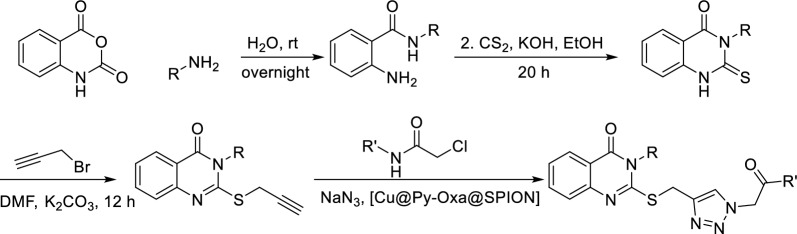
Synthesis of 3-alkyl-2-(((4-(2-oxopropyl)-1*H*-1,2,3-triazol-1-yl)alkyl)thio)-2,3-dihydroquinazolin-4(1*H*)-one products using Cu@Py-Oxa@SPION catalyst

For the synthesis of the products, 3-alkyl-2-(prop-2-yn-1-ylthio)quinazolin-4(3*H*)-one (3 mmol) was added to ethanol (10 mL), and 2-chloro-*N*-alkylacetamide (3 mmol), sodium azide (3 mmol), and Cu@Py-Oxa@SPION catalyst (50 mg) as added. The reaction mixture was stirred at room temperature and after the reaction was completed, the catalyst was isolated from the reaction mixture by a magnet. The solvent was removed and the product was purified by recrystallization from ethanol. The synthesis steps are presented in Scheme 4.

### Spectral data of the products

#### *N*-(2,4-Dimethylphenyl)-2-(4-(((3-isopropyl-4-oxo-3,4-dihydroquinazolin-2-yl)thio)methyl)-1*H*-1,2,3-triazol-1-yl)acetamide

M.p. = 195–197 °C; IR (KBr): υ = 3168, 3126, 3084, 1665, 1601, 1241 cm^−1^; ^1^H NMR (500 MHz, DMSO-*d*_6_) δ 9.72 (s, 1H), 8.15 (s, 1H), 8.03 (d, *J* = 7.9 Hz, 1H), 7.76 (t, *J* = 7.5 Hz, 1H), 7.60 (d, *J* = 8.1 Hz, 1H), 7.42 (t, *J* = 7.5 Hz, 1H), 7.04 (d, *J* = 3.8 Hz, 3H), 5.31 (s, 2H), 4.59 (s, 3H), 2.09 (s, 6H), 1.53 (d, *J* = 6.6 Hz, 6H); ^13^C NMR (126 MHz, DMSO-*d*_6_) δ 164.42, 161.07, 156.70, 146.67, 135.46, 134.99, 134.26, 128.17, 127.58, 127.21, 127.18, 126.56, 126.38, 126.23, 123.25, 119.92, 52.82, 46.07, 27.62, 19.56, 18.43. *Anal*. calcd. For C_24_H_26_N_6_O_2_S: C, 62.32; H, 5.67; N, 18.17; S, 6.93. Found: C, 62.65; H, 5.39; N, 18.03; S, 7.14; MS (70 eV): m/z = 462 (M^+^).

#### *N*-(3-Chlorophenyl)-2-(4-(((3-isopropyl-4-oxo-3,4-dihydroquinazolin-2-yl)thio)methyl)-1*H*-1,2,3-triazol-1-yl)acetamide

M.p. = 214–215 °C; IR (KBr): υ = 3170, 3128, 3086, 1667, 1601, 1240 cm^−1^; ^1^H NMR (500 MHz, DMSO-*d*_6_) δ 9.72 (s, 1H), 8.15 (s, 1H), 8.03 (d, *J* = 7.9 Hz, 1H), 7.76 (t, *J* = 7.5 Hz, 1H), 7.60 (d, *J* = 8.1 Hz, 1H), 7.42 (t, *J* = 7.5 Hz, 1H), 7.04 (d, *J* = 3.8 Hz, 3H), 5.31 (s, 2H), 4.59 (s, 3H), 2.09 (s, 6H), 1.53 (d, *J* = 6.6 Hz, 6H); ^13^C NMR (126 MHz, DMSO-*d*_6_) δ 164.98, 161.41, 156.12, 146.67, 140.22, 134.92, 133.62, 131.06, 127.16, 126.60, 126.53, 126.35, 126.28, 123.93, 120.40, 119.13, 118.03, 52.78, 45.81, 27.46, 19.56. *Anal*. calcd. For C_22_H_21_ClN_6_O_2_S: C, 65.35; H, 4.51; N, 17.92; S, 6.84. Found: C, 65.17; H, 4.72; N, 18.08; S, 6.99; MS (70 eV): m/z = 468 (M^+^).

#### *N*-(4-Bromophenyl)-2-(4-(((3-isopropyl-4-oxo-3,4-dihydroquinazolin-2-yl)thio)methyl)-1*H*-1,2,3-triazol-1-yl)acetamide

M.p. = 231–233 °C; IR (KBr): υ = 3175, 3136, 3084, 1666, 1602, 1243 cm^−1^; ^1^H NMR (500 MHz, DMSO-*d*_6_) δ 10.61 (s, 1H), 8.22 (s, 1H), 8.05 (d, *J* = 7.8 Hz, 1H), 7.76 (t, *J* = 7.6 Hz, 1H), 7.62 (d, *J* = 8.1 Hz, 1H), 7.58–7.46 (m, 4H), 7.43 (t, *J* = 7.5 Hz, 1H), 5.33 (s, 2H), 4.62 (s, 3H), 1.56 (d, *J* = 6.5 Hz, 6H); ^13^C NMR (126 MHz, DMSO-*d*_6_) δ 164.82, 161.44, 156.20, 146.70, 138.22, 134.93, 132.18, 126.56, 126.53, 126.37, 126.34, 126.28, 121.56, 120.42, 115.86, 52.75, 46.09, 27.50, 19.59. *Anal*. calcd. For C_22_H_21_BrN_6_O_2_S: C, 51.47; H, 4.12; N, 16.37; S, 6.24. Found: C, 51.74; H, 3.97; N, 16.19; S, 6.07; MS (70 eV): m/z = 512 (M^+^).

#### 2-(4-(((3-Isopropyl-4-oxo-3,4-dihydroquinazolin-2-yl)thio)methyl)-1*H*-1,2,3-triazol-1-yl)-*N*-(4-methylbenzyl)acetamide

M.p. = 208–210 °C; IR (KBr): υ = 3172, 3130, 3088, 1669, 1603, 1242 cm^−1^; ^1^H NMR (500 MHz, DMSO-*d*_6_) δ 8.75 (t, *J* = 5.9 Hz, 1H), 8.12 (s, 1H), 8.04 (dd, *J* = 8.0, 1.5 Hz, 1H), 7.76 (td, *J* = 7.7, 7.1, 1.6 Hz, 1H), 7.60 (d, *J* = 8.1 Hz, 1H), 7.42 (t, *J* = 7.6 Hz, 1H), 7.11 (q, *J* = 8.1 Hz, 3H), 5.12 (s, 2H), 4.58 (s, 3H), 4.24 (d, *J* = 5.8 Hz, 2H), 2.25 (s, 3H), 1.54 (d, *J* = 6.6 Hz, 6H); ^13^C NMR (126 MHz, DMSO-*d*_6_) δ 165.73, 161.42, 156.24, 146.69, 136.51, 136.07, 134.96, 129.30, 127.81, 126.56, 126.34, 126.24, 126.07, 126.04, 120.42, 52.09, 47.67, 42.55, 27.47, 21.10, 19.56. *Anal*. calcd. For C_24_H_26_N_6_O_2_S: C, 62.32; H, 5.67; N, 18.17; S, 6.93. Found: C, 62.16; H, 5.89; N, 18.35; S, 7.16; MS (70 eV): m/z = 462 (M^+^).

#### 2-(4-(((3-Isopropyl-4-oxo-3,4-dihydroquinazolin-2-yl)thio)methyl)-1*H*-1,2,3-triazol-1-yl)-*N*-(4-nitrophenyl)acetamide

M.p. = 198–201 °C; IR (KBr): υ = 3168, 3134, 3088, 1669, 1602, 1238 cm^−1^; ^1^H NMR (500 MHz, DMSO-*d*_6_) δ 11.08 (s, 1H), 8.24 (d, *J* = 9.1 Hz, 2H), 8.21 (s, 1H), 8.05 (dd, *J* = 8.0, 1.5 Hz, 1H), 7.83–7.75 (m, 3H), 7.63 (d, *J* = 8.0 Hz, 1H), 7.48–7.40 (m, 1H), 5.40 (s, 2H), 4.62 (s, 3H), 1.56 (d, *J* = 6.6 Hz, 6H); ^13^C NMR (126 MHz, DMSO-*d*_6_) δ 165.81, 161.45, 156.23, 146.70, 144.95, 143.00, 135.01, 131.63, 128.25, 126.57, 126.55, 126.40, 126.31, 125.60, 119.44, 52.78, 48.62, 27.44, 19.59. *Anal*. calcd. For C_22_H_21_N_7_O_4_S: C, 55.11; H, 4.41; N, 20.45; S, 6.69. Found: C, 54.95; H, 4.24; N, 20.29; S, 6.88; MS (70 eV): m/z = 479 (M^+^).

#### 2-(4-(((3-Isopropyl-4-oxo-3,4-dihydroquinazolin-2-yl)thio)methyl)-1*H*-1,2,3-triazol-1-yl)-*N*-phenylacetamide

M.p. = 212–215 °C; IR (KBr): υ = 3174, 3132, 3089, 1671, 1605, 1243 cm^−1^; ^1^H NMR (500 MHz, DMSO-*d*_6_) δ 10.43 (s, 1H), 8.19 (s, 1H), 8.03 (d, *J* = 7.9 Hz, 1H), 7.76 (t, *J* = 7.7 Hz, 1H), 7.61 (d, *J* = 7.7 Hz, 1H), 7.54 (d, *J* = 8.0 Hz, 1H), 7.42 (t, *J* = 7.5 Hz, 1H), 7.30 (t, *J* = 7.7 Hz, 2H), 7.05 (dd, *J* = 11.6, 5.4 Hz, 2H), 5.29 (s, 2H), 4.60 (s, 3H), 1.54 (d, *J* = 6.5 Hz, 6H); ^13^C NMR (126 MHz, DMSO-*d*_6_) δ 164.57, 161.43, 156.22, 146.69, 138.82, 135.47, 134.96, 129.34, 128.17, 127.18, 126.55, 126.36, 126.27, 124.19, 119.61, 52.70, 48.79, 27.49, 19.58. *Anal*. calcd. For C_22_H_22_N_6_O_2_S: C, 60.81; H, 5.10; N, 19.34; S, 7.38. Found: C, 60.98; H, 5.22; N, 19.56; S, 7.47; MS (70 eV): m/z = 434 (M^+^).

#### *N*-(3,5-Dimethylphenyl)-2-(4-(((4-oxo-3-phenyl-3,4-dihydroquinazolin-2-yl)thio)methyl)-1*H*-1,2,3-triazol-1-yl)acetamide

M.p. = 206–208 °C; IR (KBr): υ = 3171, 3128, 3086, 1666, 1601, 1243 cm^−1^; ^1^H NMR (500 MHz, DMSO-*d*_6_) δ 9.72 (s, 1H), 8.13 (s, 1H), 8.09 (d, *J* = 8.0 Hz, 1H), 7.84 (t, *J* = 7.9 Hz, 1H), 7.71 (d, *J* = 8.2 Hz,  H), 7.59–7.36 (m, 7H), 7.05 (s, 3H), 5.31 (s, 2H), 4.48 (s, 2H), 2.11 (s, 6H); ^13^C NMR (126 MHz, DMSO-*d*_6_) δ 164.42, 161.16, 157.12, 147.67, 136.16, 135.49, 135.37, 134.60, 132.17, 130.39, 129.95, 129.85, 128.18, 127.19, 127.02, 126.67, 126.51, 126.02, 120.05, 52.12, 27.28, 18.45. *Anal*. calcd. For C_27_H_24_N_6_O_2_S: C, 65.30; H, 4.87; N, 16.92; S, 4.46. Found: C, 65.12; H, 4.69; N, 17.11; S, 4.25; MS (70 eV): m/z = 496 (M^+^).

#### *N*-(3-Chlorophenyl)-2-(4-(((4-oxo-3-phenyl-3,4-dihydroquinazolin-2-yl)thio)methyl)-1*H*-1,2,3-triazol-1-yl)acetamide

M.p. = 221–223 °C; IR (KBr): υ = 3175, 3134, 3091, 1672, 1606, 1245 cm^−1^; ^1^H NMR (500 MHz, DMSO-*d*_6_) δ 10.67 (s, 1H), 8.18 (s, 1H), 8.10 (d, *J* = 7.9 Hz, 1H), 7.79–7.70 (m, 2H), 7.61–7.39 (m, 9H), 7.15 (d, *J* = 8.3 Hz, 1H), 5.31 (s, 2H), 4.50 (s, 2H); ^13^C NMR (126 MHz, DMSO-*d*_6_) δ 165.09, 161.19, 157.12, 147.69, 140.26, 136.19, 135.38, 134.61, 133.65, 131.11, 130.43, 129.99, 129.88, 129.74, 127.02, 126.74, 126.53, 123.97, 120.07, 119.15, 118.06, 52.67, 27.31. *Anal*. calcd. For C_25_H_19_ClN_6_O_2_S: C, 59.70; H, 3.81; N, 16.71; S, 6.37. Found: C, 59.92; H, 4.01; N, 16.53; S, 6.20; MS (70 eV): m/z = 502 (M^+^).

#### *N*-(4-Bromophenyl)-2-(4-(((4-oxo-3-phenyl-3,4-dihydroquinazolin-2-yl)thio)methyl)-1*H*-1,2,3-triazol-1-yl)acetamide

M.p. = 185–187 °C; IR (KBr): υ = 3173, 3131, 3087, 1669, 1602, 1244 cm^−1^; ^1^H NMR (500 MHz, DMSO-*d*_6_) δ 10.57 (s, 1H), 8.12 (s, 1H), 8.08 (d, *J* = 7.8 Hz, 1H), 7.84 (t, *J* = 7.7 Hz, 1H), 7.71 (d, *J* = 8.1 Hz, 1H), 7.60–7.40 (m, 10H), 5.27 (s, 2H), 4.47 (s, 2H); ^13^C NMR (126 MHz, DMSO-*d*_6_) δ 164.82, 161.16, 157.11, 147.67, 138.19, 136.16, 135.37, 132.18, 130.40, 129.96, 129.86, 127.01, 126.71, 126.51, 126.10, 125.56, 121.56, 120.05, 115.83, 52.64, 27.27. *Anal*. calcd. For C_25_H_19_BrN_6_O_2_S: C, 54.85; H, 3.50; N, 15.35; S, 5.86. Found: C, 55.02; H, 3.74; N, 15.59; S, 6.01; MS (70 eV): m/z = 546 (M^+^).

#### *N*-(4-Nitrophenyl)-2-(4-(((4-oxo-3-phenyl-3,4-dihydroquinazolin-2-yl)thio)methyl)-1*H*-1,2,3-triazol-1-yl)acetamide

M.p. = 231–233 °C; IR (KBr): υ = 3172, 3132, 3089, 1670, 1603, 1244 cm^−1^; ^1^H NMR (500 MHz, DMSO-*d*_6_) δ 11.04 (s, 1H), 8.28–8.11 (m, 3H), 8.07 (d, *J* = 7.8 Hz, 1H), 7.88–7.66 (m, 5H), 7.60–7.38 (m, 6H), 5.36 (s, 2H), 4.48 (s, 2H); ^13^C NMR (126 MHz, DMSO-*d*_6_) δ 165.73, 161.15, 157.08, 147.66, 144.91, 142.99, 136.16, 135.36, 131.97, 130.40, 129.96, 129.85, 128.73, 127.00, 126.71, 126.50, 125.53, 120.03, 119.43, 52.78, 27.32; *Anal*. calcd. For C_25_H_19_N_7_O_4_S: C, 58.47; H, 3.73; N, 19.09; S, 6.24. Found: C, 58.27; H, 3.66; N, 18.88; S, 6.06; MS (70 eV): m/z = 513 (M^+^).

#### 2-(4-(((4-Oxo-3-phenyl-3,4-dihydroquinazolin-2-yl)thio)methyl)-1*H*-1,2,3-triazol-1-yl)-*N*-phenylacetamide

M.p. = 224–225 °C; IR (KBr): υ = 3167, 3127, 3086, 1665, 1602, 1241 cm^−1^; ^1^H NMR (500 MHz, DMSO-*d*_6_) δ 10.44 (s, 1H), 8.15 (s, 1H), 8.08 (d, *J* = 7.8 Hz, 1H), 7.83 (t, *J* = 7.8 Hz, 1H), 7.71 (d, *J* = 8.1 Hz, 1H), 7.59–7.49 (m, 5H), 7.50–7.40 (m, 3H), 7.30 (t, *J* = 7.8 Hz, 2H), 7.06 (t, *J* = 7.4 Hz, 1H), 5.27 (s, 2H), 4.48 (s, 2H); ^13^C NMR (126 MHz, DMSO-*d*_6_) δ 164.57, 161.17, 157.10, 147.67, 138.82, 136.16, 135.37, 130.40, 129.96, 129.86, 129.35, 127.00, 126.71, 126.50, 124.20, 120.04, 119.60, 52.67, 27.29; *Anal*. calcd. For C_25_H_20_N_6_O_2_S: C, 64.09; H, 4.30; N, 17.94; S, 6.84. Found: C, 63.90; H, 4.51; N, 17.81; S, 7.03; MS (70 eV): m/z = 468 (M^+^).

#### 2-(4-(((3-Benzyl-4-oxo-3,4-dihydroquinazolin-2-yl)thio)methyl)-1*H*-1,2,3-triazol-1-yl)-*N*-(2,4-dimethylphenyl)acetamide

M.p. = 219–221 °C; IR (KBr): υ = 3172, 3128, 3086, 1667, 1602, 1241 cm^−1^; ^1^H NMR (500 MHz, DMSO-*d*_6_) δ 9.72 (s, 1H), 8.12 (s, 1H), 7.82 (t, *J* = 7.7 Hz, 1H), 7.67 (d, *J* = 8.1 Hz, 1H), 7.48 (t, *J* = 7.5 Hz, 1H), 7.34–7.18 (m, 6H), 7.04 (q, *J* = 5.0 Hz, 3H), 5.31 (s, 2H), 5.29 (s, 2H), 4.59 (s, 2H), 2.09 (s, 6H); ^13^C NMR (126 MHz, DMSO-*d*_6_) δ 164.39, 161.37, 156.70, 147.26, 136.00, 135.47, 135.39, 134.59, 130.28, 129.04, 128.17, 127.85, 127.18, 127.13, 127.06, 126.63, 126.11, 119.43, 119.20, 52.13, 47.22, 27.06, 18.43. *Anal*. calcd. For C_28_H_26_N_6_O_2_S: C, 65.86; H, 5.13; N, 16.46; S, 6.28. Found: C, 65.63; H, 5.00; N, 16.31; S, 6.12; MS (70 eV): m/z = 510 (M^+^).

#### 2-(4-(((3-Benzyl-4-oxo-3,4-dihydroquinazolin-2-yl)thio)methyl)-1*H*-1,2,3-triazol-1-yl)-*N*-(4-bromophenyl)acetamide

M.p. = 197–199 °C; IR (KBr): υ = 3174, 3132, 3091, 1670, 1604, 1243 cm^−1^; ^1^H NMR (500 MHz, DMSO-*d*_6_) δ 10.56 (s, 1H), 8.14–8.09 (m, 2H), 7.83 (t, *J* = 7.3 Hz, 1H), 7.69 (d, *J* = 7.7 Hz, 1H), 7.55–7.45 (m, 5H), 7.30 (t, *J* = 8.0 Hz, 2H), 7.23 (dd, *J* = 14.0, 7.4 Hz, 3H), 5.30 (s, 2 ), 5.27 (s, 2H), 4.59 (s, 2H); ^13^C NMR (126 MHz, DMSO-*d*_6_) δ 164.82, 161.38, 156.69, 147.27, 136.01, 135.40, 134.81, 133.70, 132.18, 130.15, 129.05, 127.86, 127.13, 127.05, 126.69, 126.66, 126.11, 121.55, 119.20, 52.64, 47.23, 27.00. *Anal*. calcd. For C_26_H_21_BrN_6_O_2_S: C, 55.62; H, 3.77; N, 14.97; S, 5.71. Found: C, 55.85; H, 3.47; N, 15.18; S, 5.56; MS (70 eV): m/z = 560 (M^+^).

#### 2-(4-(((3-Benzyl-4-oxo-3,4-dihydroquinazolin-2-yl)thio)methyl)-1*H*-1,2,3-triazol-1-yl)-*N*-(4-nitrophenyl)acetamide

M.p. = 212–214 °C; IR (KBr): υ = 3172, 3130, 3088, 1669, 1603, 1242 cm^−1^; ^1^H NMR (500 MHz, DMSO-*d*_6_) δ 11.08 (s, 1H), 8.23 (d, *J* = 9.0 Hz, 2H), 8.17 (s, 1H), 8.12 (d, *J* = 7.9 Hz, 1H), 7.81 (dd, *J* = 11.4, 8.2 Hz, 3H), 7.69 (d, *J* = 8.2 Hz, 1H), 7.49 (t, *J* = 7.6 Hz, 1H), 7.28 (dt, *J* = 35.0, 7.5 Hz, 5H), 5.39 (s, 2H), 5.31 (s, 2H), 4.62 (s, 2H); ^13^C NMR (126 MHz, DMSO-*d*_6_) δ 165.77, 161.38, 156.67, 147.27, 144.94, 143.00, 136.02, 135.37, 131.23, 129.06, 128.82, 127.87, 127.16, 127.05, 126.68, 126.63, 125.54, 119.43, 119.20, 52.78, 47.25, 27.06. *Anal*. calcd. For C_26_H_21_N_7_O_4_S: C, 59.19; H, 4.01; N, 18.59; S, 6.08. Found: C, 59.33; H, 3.89; N, 18.77; S, 5.91; MS (70 eV): m/z = 527 (M^+^).

#### 2-(4-(((3-Benzyl-4-oxo-3,4-dihydroquinazolin-2-yl)thio)methyl)-1*H*-1,2,3-triazol-1-yl)-*N*-phenylacetamide

M.p. = 224–225 °C; IR (KBr): υ = 3168, 3126, 3087, 1666, 1600, 1241 cm^−1^; ^1^H NMR (500 MHz, DMSO-*d*_6_) δ 10.45 (s, 1H), 8.16 (s, 1H), 8.11 (d, *J* = 7.9 Hz, 1H), 7.80 (t, *J* = 7.7 Hz, 1H), 7.67 (d, *J* = 8.1 Hz, 1H), 7.56 (d, *J* = 8.0 Hz, 2H), 7.46 (t, *J* = 7.6 Hz, 1 H), 7.26 (dd, *J* = 31.9, 8.1 Hz, 8H), 7.06 (t, *J* = 7.4 Hz, 1H), 5.30 (s, 4H), 4.61 (s, 2H); ^13^C NMR (126 MHz, DMSO-*d*_6_) δ 164.57, 161.37, 156.66, 147.26, 138.84, 136.01, 135.33, 129.33, 129.03, 128.81, 127.85, 127.17, 127.04, 126.66, 126.59, 126.23, 124.19, 119.63, 119.21, 52.71, 47.24, 27.10. *Anal*. calcd. For C_26_H_22_N_6_O_2_S: C, 64.71; H, 4.60; N, 17.42; S, 6.64. Found: C, 46.56; H, 4.82; N, 17.26; S, 6.86; MS (70 eV): m/z = 482 (M^+^).

#### 2-(4-(((3-Benzyl-4-oxo-3,4-dihydroquinazolin-2-yl)thio)methyl)-1*H*-1,2,3-triazol-1-yl)-*N*-(3-chlorophenyl)acetamide

M.p. = 238–239 °C; IR (KBr): υ = 3171, 3131, 3088, 1668, 1603, 1242 cm^−1^; ^1^H NMR (500 MHz, DMSO-*d*_6_) δ 10.66 (s, 1H), 8.20 (s, 1H), 8.12 (d, *J* = 7.8 Hz, 1H), 7.82 (t, *J* = 7.4 Hz, 1H), 7.76 (s, 1H), 7.69 (d, *J* = 8.1 Hz, 1H), 7.48 (t, *J* = 7.5 Hz, 1H), 7.42 (d, *J* = 8.1 Hz, 1H), 7.33 (dt, *J* = 18.5, 7.6 Hz, 4H), 7.24 (d, *J* = 7.6 Hz, 3H), 7.14 (d, *J* = 7.9 Hz, 1H), 5.32 (d, *J* = 13.3 Hz, 4H), 4.62 (s, 2H); ^13^C NMR (126 MHz, DMSO-d6) δ 165.01, 161.38, 156.60, 147.26, 140.24, 136.01, 135.34, 133.65, 131.07, 129.05, 127.86, 127.18, 127.05, 126.69, 126.63, 123.95, 119.22, 119.15, 118.04, 52.76, 47.25, 27.06. *Anal*. calcd. For C_26_H_21_ClN_6_O_2_S: C, 60.40; H, 4.09; N, 16.26; S, 6.20. Found: C, 60.23; H, 3.85; N, 16.05; S, 5.96; MS (70 eV): m/z = 516 (M^+^).

### Supplementary Information

**Additional file 1:** Images of ^1^H NMR and ^13^C NMR of the new synthesized compounds are available in the Supporting Information.

## Data Availability

The datasets used and/or analysed during the current study available from the corresponding author on reasonable request.

## References

[1] Kamel S, Khattab TA (2021). Recent advances in cellulose supported metal nanoparticles as green and sustainable catalysis for organic synthesis. Cellulose.

[2] Bordet A, Leitner W (2021). Metal nanoparticles immobilized on molecularly modified surfaces: versatile catalytic systems for controlled hydrogenation and hydrogenolysis. Acc Chem Res.

[3] Shafiei N, Nasrollahzadeh M, Iravani S (2021). Green synthesis of silica and silicon nanoparticles and their biomedical and catalytic applications. Comments Inorg Chem.

[4] Sayahi MH, Toosibashi M, Bahmaei M, Lijan H, Ma'Mani L, Mahdavi M, Bahadorikhalili S (2022). Pd@ Py2PZ@ MSN as a novel and efficient catalyst for CC bond formation reactions. Front Chem.

[5] Ghorbani-Choghamarani A, Mohammadi M, Tamoradi T, Ghadermazi M (2019). Covalent immobilization of Co complex on the surface of SBA-15: green, novel and efficient catalyst for the oxidation of sulfides and synthesis of polyhydroquinoline derivatives in green condition. Polyhedron.

[6] Esmaili S, Khazaei A, Ghorbani-Choghamarani A, Mohammadi M (2022). Silica sulfuric acid coated on SnFe_2_O_4_ MNPs: synthesis, characterization and catalytic applications in the synthesis of polyhydroquinolines. RSC Adv.

[7] Mohammadi M, Ghorbani-Choghamarani A (2022). Hercynite silica sulfuric acid: a novel inorganic sulfurous solid acid catalyst for one-pot cascade organic transformations. RSC Adv.

[8] Mohammadi M, Khodamorady M, Tahmasbi B, Bahrami K, Ghorbani-Choghamarani A (2021). Boehmite nanoparticles as versatile support for organic–inorganic hybrid materials: synthesis, functionalization, and applications in eco-friendly catalysis. J Ind Eng Chem.

[9] Kazemi M, Mohammadi M (2020). Magnetically recoverable catalysts: catalysis in synthesis of polyhydroquinolines. Appl Organomet Chem.

[10] Mokhtary M (2016). Recent advances in catalysts immobilized on magnetic nanoparticles. J Iran Chem Soc.

[11] Arya I, Poona A, Dikshit PK, Pandit S, Kumar J, Singh HN, Jha NK, Rudayni HA, Chaudhary AA, Kumar S (2021). Current trends and future prospects of nanotechnology in biofuel production. Catalysts.

[12] Sayahi MH, Ghomi M, Hamad SM, Ganjali MR, Aghazadeh M, Mahdavi M, Bahadorikhalili S (2021). Electrochemical synthesis of three-dimensional flower‐like Ni/Co–BTC bimetallic organic framework as heterogeneous catalyst for solvent‐free and green synthesis of substituted chromeno[4,3-*b*]quinolones. J Chin Chem Soc.

[13] Fatahi Y, Ghaempanah A, Ma'mani L, Mahdavi M, Bahadorikhalili S (2021). Palladium supported aminobenzamide modified silica coated superparamagnetic iron oxide as an applicable nanocatalyst for heck cross-coupling reaction. J Organomet Chem.

[14] Mohammadi M, Ghorbani-Choghamarani A (2022). A novel hercynite-supported tetradentate Schiff base complex of manganese catalyzed one-pot annulation reactions. Appl Organomet Chem.

[15] Mohammadi M, Ghorbani-Choghamarani A (2022). Synthesis and characterization of novel hercynite@sulfuric acid and its catalytic applications in the synthesis of polyhydroquinolines and 2,3-dihydroquinazolin-4(1*H*)-ones. RSC Adv.

[16] Ghobakhloo F, Azarifar D, Mohammadi M, Keypour H, Zeynali H (2022). Copper(II) Schiff-base complex modified UiO-66-NH_2_(Zr) metal–organic framework catalysts for Knoevenagel condensation–Michael addition–cyclization reactions. Inorg Chem.

[17] Riadi Y, Kadhim M, Jawad Shoja M, Hussein Ali S, Fakri Mustafa M, Sajjadi Y (2022). Copper (II) complex supported on the surface of magnetic nanoparticles modified with *S*-benzylisothiourea (Fe_3_O_4_@SiO_2_-SMTU-Cu): a new and efficient nanomagnetic catalyst for the synthesis of quinazolines and amides. Synth Commun.

[18] Abdolmohammadi S, Shariati S, Fard NE, Samani A (2020). Aqueous-mediated green synthesis of novel spiro[indole‐quinazoline]derivatives using kit‐6 mesoporous silica coated Fe_3_O_4_ nanoparticles as catalyst. J Heterocycl Chem.

[19] Marais L, Vosloo HC, Swarts AJ (2021). Homogeneous oxidative transformations mediated by copper catalyst systems. Coord Chem Rev.

[20] Cheng L-J, Mankad NP (2021). Copper-catalyzed carbonylative coupling of alkyl halides. Acc Chem Res.

[21] Sadegh Asgari M, Bahadorikhalili S, Rahimi R, Mahdavi M (2021). Copper supported onto magnetic nanoparticles as an efficient catalyst for the synthesis of triazolobenzodiazepino [7,1-*b*]quinazolin‐11(9*H*)‐ones via click *N*‐arylation reactions. ChemistrySelect.

[22] Ding Y, Fu L, Peng X, Lei M, Wang C, Jiang J (2022). Copper catalysts for radical and nonradical persulfate based advanced oxidation processes: certainties and uncertainties. Chem Eng J.

[23] Wang L, Jiang J, Ma J, Pang S, Zhang T (2022). A review on advanced oxidation processes homogeneously initiated by copper (II). Chem Eng J.

[24] Martos M, Pastor IM (2022). Iron-based imidazolium salt as dual Lewis acid and redox catalyst for the aerobic synthesis of quinazolines. Eur J Org Chem.

[25] Bui JC, Kim C, Weber AZ, Bell AT (2021). Dynamic boundary layer simulation of pulsed CO_2_ electrolysis on a copper catalyst. ACS Energy Lett.

[26] Alossaimi MA, Riadi Y, Geesi MH, Aldhafiri MK, Alanazi AI, Dehbi O, Ibnouf EO, Azzallou R (2022). Characterization, biological evaluation and molecular docking of a synthesised quinazolinone-based derivative. J Mol Struct.

[27] Li J, Ozden A, Wan M, Hu Y, Li F, Wang Y, Zamani RR, Ren D, Wang Z, Xu Y (2021). Silica-copper catalyst interfaces enable carbon-carbon coupling towards ethylene electrosynthesis. Nat Commun.

[28] Salih KS (2022). Modern development in copper-and nickel-catalyzed cross-coupling reactions: formation of carbon-carbon and carbon-heteroatom bonds under microwave irradiation conditions. Asian J Org Chem.

[29] Tamoradi T, Masoumeh Mousavi S, Mohammadi M (2020). C–C and C–S coupling catalyzed by supported Cu(II) on nano CoFe_2_O_4_. ChemistrySelect.

[30] Rezaei-Ghaleh N, Agudo-Canalejo J, Griesinger C, Golestanian R (2022). Molecular diffusivity of click reaction components: the diffusion enhancement question. J Am Chem Soc.

[31] Bahadorikhalili S, Ma’mani L, Mahdavi H, Shafiee A (2018). Copper supported β-cyclodextrin functionalized PEGylated mesoporous silica nanoparticle-graphene oxide hybrid: an efficient and recyclable nano-catalyst for straightforward synthesis of 2-arylbenzimidazoles and 1,2,3-triazoles. Microporous Mesoporous Mater.

[32] Bahadorikhalili S, Ashtari A, Ma’Mani L, Ranjbar PR, Mahdavi M (2018). Copper-supported β-cyclodextrin-functionalized magnetic nanoparticles: efficient multifunctional catalyst for one-pot ‘green’ synthesis of 1,2,3-triazolylquinazolinone derivatives. Appl Organomet Chem.

[33] Asgari MS, Bahadorikhalili S, Ghaempanah A, Ranjbar PR, Rahimi R, Abbasi A, Larijani B, Mahdavi M (2021). Copper-catalyzed one-pot synthesis of amide linked 1,2,3-triazoles bearing aryloxy skeletons. Tetrahedron Lett.

[34] Liang L, Astruc D (2011). The copper (I)-catalyzed alkyne-azide cycloaddition (CuAAC)click reaction and its applications. An overview. Coord Chem Rev.

[35] Mohammadi M, Ghorbani-Choghamarani A (2022). Complexation of guanidino containing l-arginine with nickel on silica-modified hercynite MNPs: a novel catalyst for the Hantzsch synthesis of polyhydroquinolines and 2,3-dihydroquinazolin-4(1*H*)-ones. Res Chem Intermed.

[36] Sadeghi Meresht A, Ezzatzadeh E, Dehbandi B, Salimifard M, Rostamian R (2022). Fe_3_O_4_/CuO nanocomposite promoted green synthesis of functionalized quinazolines using water extract of lettuce leaves as green media: study of antioxidant activity. Polycycl Aromat Compd.

[37] Cunha AC, Figueiredo JM, Tributino JL, Miranda AL, Castro HC, Zingali RB, Fraga CA, de Souza MC, Ferreira VF, Barreiro EJ (2003). Antiplatelet properties of novel *N*-substituted-phenyl-1,2,3-triazole-4-acylhydrazone derivatives. Bioorganic Med Chem.

[38] El Azab IH, Gobouri AA, Altalhi TA, El-Sheshtawy H, Almutlaq N, Maddah HA, Zoromba MS, Abdel-Aziz M, Bassyouni M, Ibrahim A (2021). Synthesis, characterization, DFT-TDDFT calculations and optical properties of a novel pyrazole-1,2,3-triazole hybrid thin film. Optik.

[39] Wei L, Sui H, Zhang J, Guo Z (2021). Synthesis and antioxidant activity of the inulin derivative bearing 1,2,3-triazole and diphenyl phosphate. Int J Biol Macromol.

[40] Tan W, Li Q, Li W, Dong F, Guo Z (2016). Synthesis and antioxidant property of novel 1,2,3-triazole-linked starch derivatives via ‘click chemistry’. Int J Biol Macromol.

[41] Kumar SV, Scottwell SØ, Waugh E, McAdam CJ, Hanton LR, Brooks HJ, Crowley JD (2016). Antimicrobial properties of tris (homoleptic) ruthenium(II) 2-pyridyl-1,2,3-triazole click complexes against pathogenic bacteria, including methicillin-resistant *Staphylococcus aureus* (MRSA). Inorg Chem.

[42] Zhang B (2019). Comprehensive review on the anti-bacterial activity of 1,2,3-triazole hybrids. Eur J Med Chem.

[43] El Malah T, Nour HF, Satti AA, Hemdan BA, El-Sayed WA (2020). Design, synthesis, and antimicrobial activities of 1,2,3-triazole glycoside clickamers. Molecules.

[44] Alam MM (2022). 1,2,3-Triazole hybrids as anticancer agents: a review. Arch Pharm.

[45] Duan Y-C, Ma Y-C, Zhang E, Shi X-J, Wang M-M, Ye X-W, Liu H-M (2013). Design and synthesis of novel 1,2,3-triazole-dithiocarbamate hybrids as potential anticancer agents. Eur J Med Chem.

[46] Pokhodylo N, Shyyka O, Matiychuk V (2014). Synthesis and anticancer activity evaluation of new 1,2,3-triazole-4-carboxamide derivatives. Med Chem Res.

[47] El-Feky S (1993). Synthesis and anticonvulsant properties of some novel quinazolinone thiazolidine and 4-thiazolidone derivatives. Pharmazie.

[48] Ubale P, Mokale S, More S, Waghamare S, More V, Munirathinam N, Dilipkumar S, Das RK, Reja S, Helavi VB (2022). Evaluation of in vitro anticancer, antimicrobial and antioxidant activities of new Cu(II) complexes derived from 4(3*H*)-quinazolinone: synthesis, crystal structure and molecular docking studies. J Mol Struct.

[49] Mohammad Hosein MH, Saghanezhad SJ, Bahadorikhalili S, Mahdavi M (2019). CuBr-catalysed one‐pot multicomponent synthesis of 3‐substituted 2‐thioxo‐2, 3‐dihydroquinazolin‐4 (1*H*)‐one derivatives. Appl Organomet Chem.

[50] Syed T, Asiri YI, Shaheen S, Gangarapu K (2021). Design, synthesis and anticancer evaluation of structurally modified substituted aryl-quinazoline derivatives as anticancer agents. Synth Commun.

[51] Heydari Z, Bahadorikhalili S, Rashidi Ranjbar P, Mahdavi M (2018). DABCO-modified super‐paramagnetic nanoparticles as an efficient and water‐compatible catalyst for the synthesis of pyrano[3,2-*c*:5,6‐*c′*]dichromene‐6,8‐dione derivatives under mild reaction conditions. Appl Organomet Chem.

[52] Markosyan A, Ayvazyan A, Gabrielyan S, Mamyan S, Arsenyan F, Safaryan A, Arakelyan H (2021). Synthesis and antitumor and antibacterial properties of 3-benzyl-spiro[benzo [h005DQuinazoline-5,1′-cycloheptane]-4(6*H*)-one derivatives. Pharm Chem J.

[53] Riadi Y, Geesi MH, Dehbi O, Ouerghi O (2022). Photocatalytic synthesis of quinazolinone derivatives as mediated by titanium dioxide (TiO_2_) nanoparticles greenly synthesised via citrus limon juice. Polycycl Aromat Compd.

[54] Elkholy AE, Rizk SA, Rashad AM (2019). Enhancing lubricating oil properties using novel quinazolinone derivatives: DFT study and molecular dynamics simulation. J Mol Struct.

[55] Keyhani A, Nikpassand M, Fekri LZ, Kefayati H (2022). Green synthesis of novel azo-linked 2-aryl-quinazolinones using of NiFe_2_O_4_@SP@GA nanoparticle. Polycycl Aromat Compd.

[56] Foroughi Kaldareh M, Mokhtary M, Nikpassand M (2021). Deep eutectic solvent mediated one-pot synthesis of hydrazinyl-4-phenyl-1, 3-thiazoles. Polycycl Aromat Compd.

[57] Patil VS, Padalkar VS, Chaudhari AS, Sekar N (2012). Intrinsic catalytic activity of an acidic ionic liquid as a solvent for quinazoline synthesis. Catal Sci Technol.

[58] Kumar KS, Kumar PM, Kumar KA, Sreenivasulu M, Jafar AA, Rambabu D, Krishna GR, Reddy CM, Kapavarapu R, Shivakumar K (2011). A new three-component reaction: green synthesis of novel isoindolo [2, 1-a] quinazoline derivatives as potent inhibitors of TNF-α. Chem Commun.

[59] Wang S-L, Yang K, Yao C-S, Wang X-S (2012). Green synthesis of quinazolinone derivatives catalyzed by iodine in ionic liquid. Synth Commun.

[60] Torabi M, Fekri LZ, Nikpassand M (2022). Synthesis, characterization and application of Fe_3_O_4_@silicapropyl@vaniline-covalented isoniazid-copper(I) nanocomposite as a new, mild, effective and magnetically recoverable Lewis acid catalyst for the synthesis of acridines and novel azoacridines. J Mol Struct.

[61] Nikpassand M, Fekri LZ, Sharafi S (2013). An efficient and green synthesis of novel azo Schiff base and its complex under ultrasound irradiation. Orient J Chem.

[62] Nikpassand M, Keyhani A, Fekri LZ, Varma RS (2022). Mechanochemical synthesis of azo-linked 2-amino-4*H*-chromene derivatives using Fe_3_O_4_@SiO_2_@KIT-6-NH_2_@Schiff-base complex nanoparticles. J Mol Struct.

[63] Nikpassand M, Fekri LZ, Pourahmad A (2018). One-pot synthesis of new azo-linked 4*H*-benzo [d][1, 3] oxazine-2, 4-diones from carbon dioxide using CuO@ RHA/MCM-41 nanocomposite in green media. J Co2 Util.

