# Supplementary material for: Efficient synthesis of 3-alkyl-2-(-1H-1,2,3-triazolyl)methyl)thio)-2,3-dihydroquinazolin-4(1H)-one derivative via multistep synthesis approach by novel Cu@Py-Oxa@SPION catalyst
Source: BMC Chem. 2023 Nov 14;17(1):154. doi: 10.1186/s13065-023-01072-4 (PMC10647046; doi:10.1186/s13065-023-01072-4)
Supplement: Supplementary file 1 — Additional file 1: Images of 1H NMR and 13C NMR of the new synthesized compounds are available in the Supporting Information. [file 13065_2023_1072_MOESM1_ESM.pdf]

Efficient Synthesis of 3-Alkyl-2-(-1*H*-1,2,3-triazolyl)methyl)thio)-2,3-dihydroquinazolin-4(1*H*)-one Derivative via Multistep synthesis approach by Novel Cu@Py-Oxa@SPION Catalyst

Alireza Sherafati[1], Shahram Moradi[1], Mohammad Mahdavi*[2]

[1] *Department of Chemistry Tehran North Branch, Islamic Azad University, Tehran, Iran.*

[2] *Endocrinology and Metabolism Research Centre, Endocrinology and Metabolism Clinical Sciences Institute, Tehran University of Medical Sciences, Tehran, Iran, email:* momahdavi@sina.tums.ac.ir.

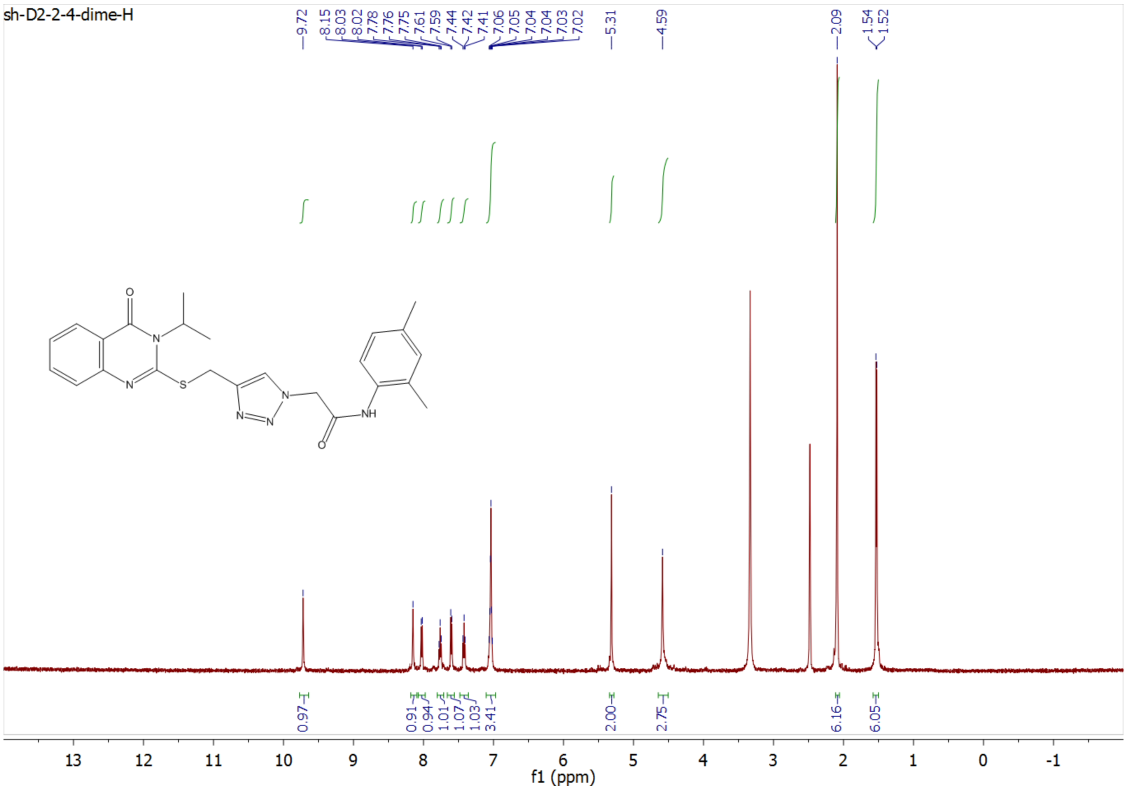

Figure S 1. ^{1}H NMR spectrum of N-(2,4-dimethylphenyl)-2-(4-(((3-isopropyl-4-oxo-3,4-dihydroquinazolin-2-yl)thio)methyl)-1H-1,2,3-triazol-1-yl)acetamide

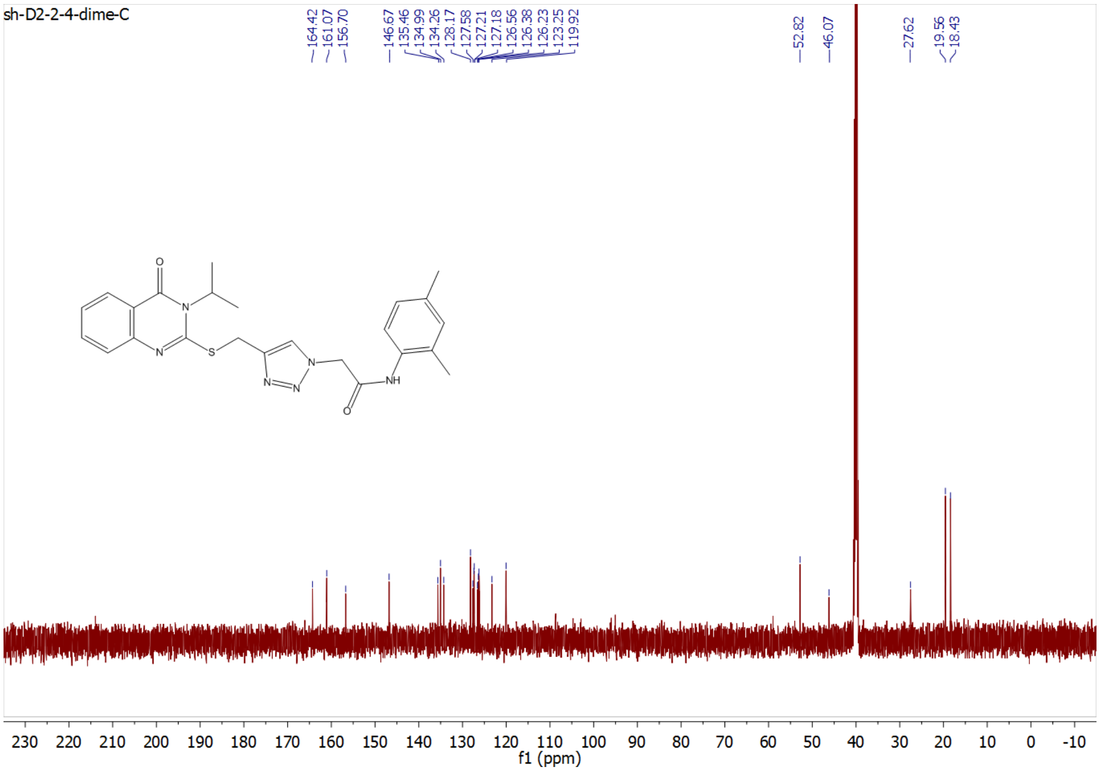

Figure S 2. ^{13}C NMR spectrum of N-(2,4-dimethylphenyl)-2-(4-(((3-isopropyl-4-oxo-3,4-dihydroquinazolin-2-yl)thio)methyl)-1H-1,2,3-triazol-1-yl)acetamide

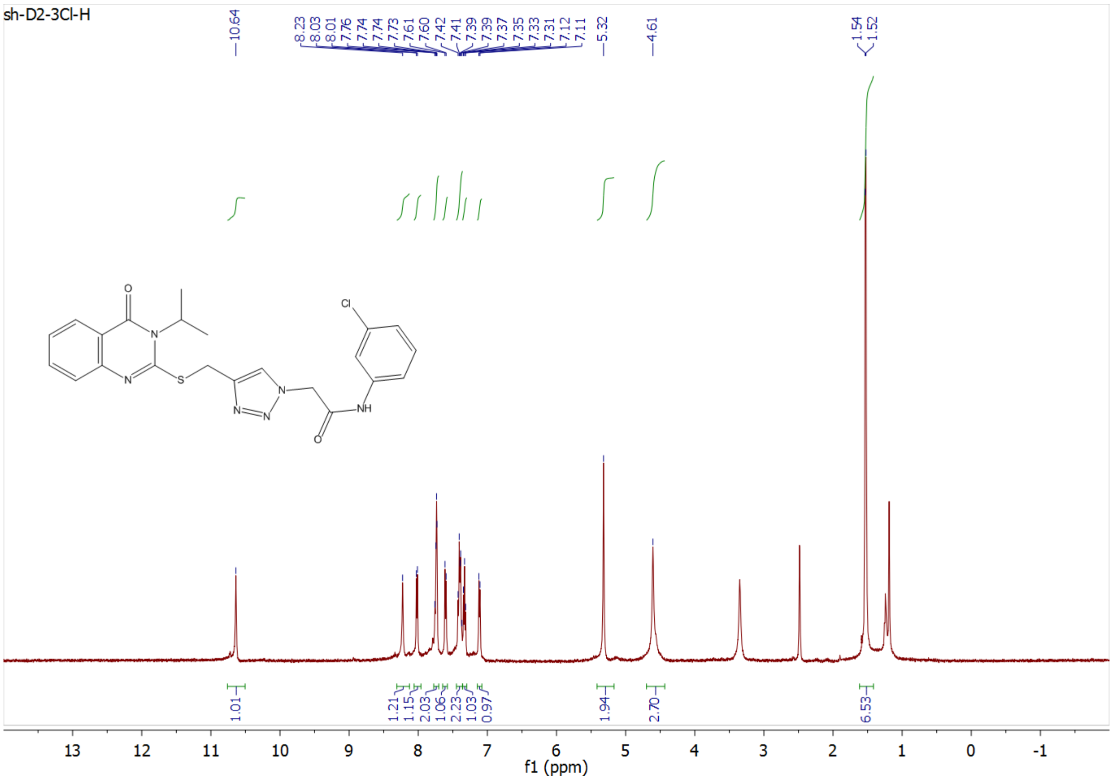

Figure S 3. ^{1}H NMR spectrum of N-(3-chlorophenyl)-2-(4-(((3-isopropyl-4-oxo-3,4-dihydroquinazolin-2-yl)thio)methyl)-1H-1,2,3-triazol-1-yl)acetamide

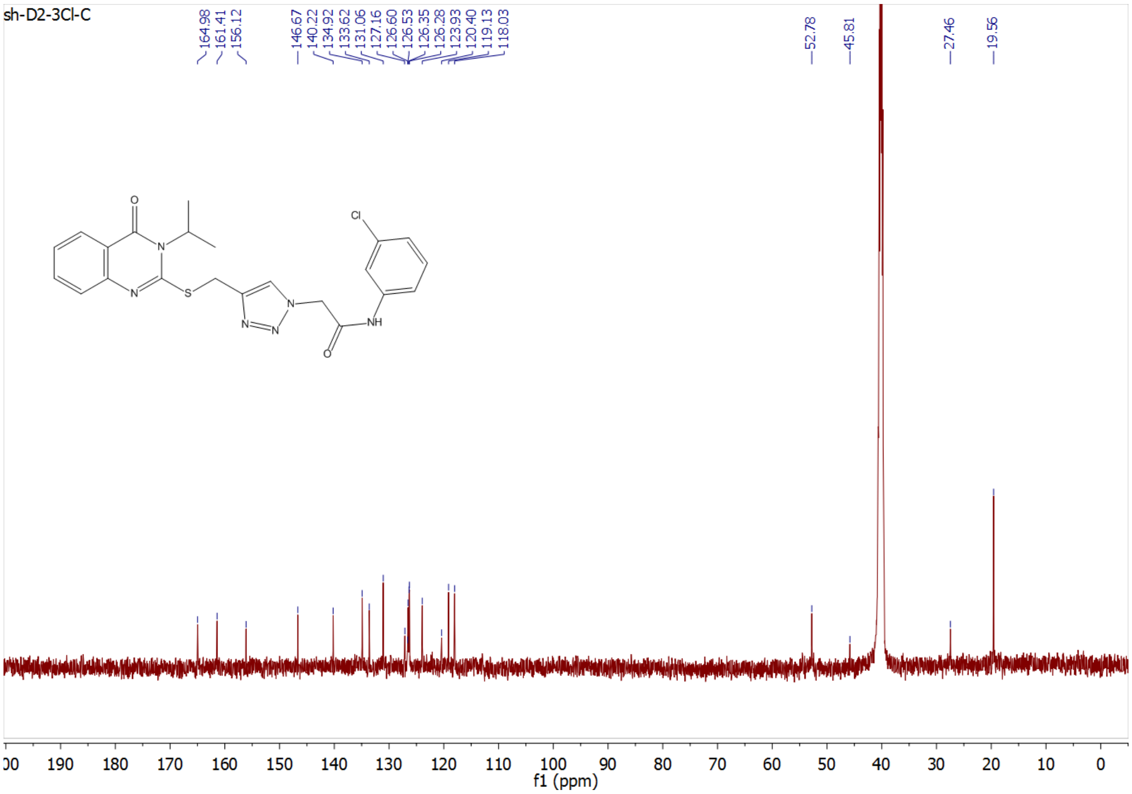

Figure S 4. ^{13}C NMR spectrum of N-(3-chlorophenyl)-2-(4-(((3-isopropyl-4-oxo-3,4-dihydroquinazolin-2-yl)thio)methyl)-1H-1,2,3-triazol-1-yl)acetamide

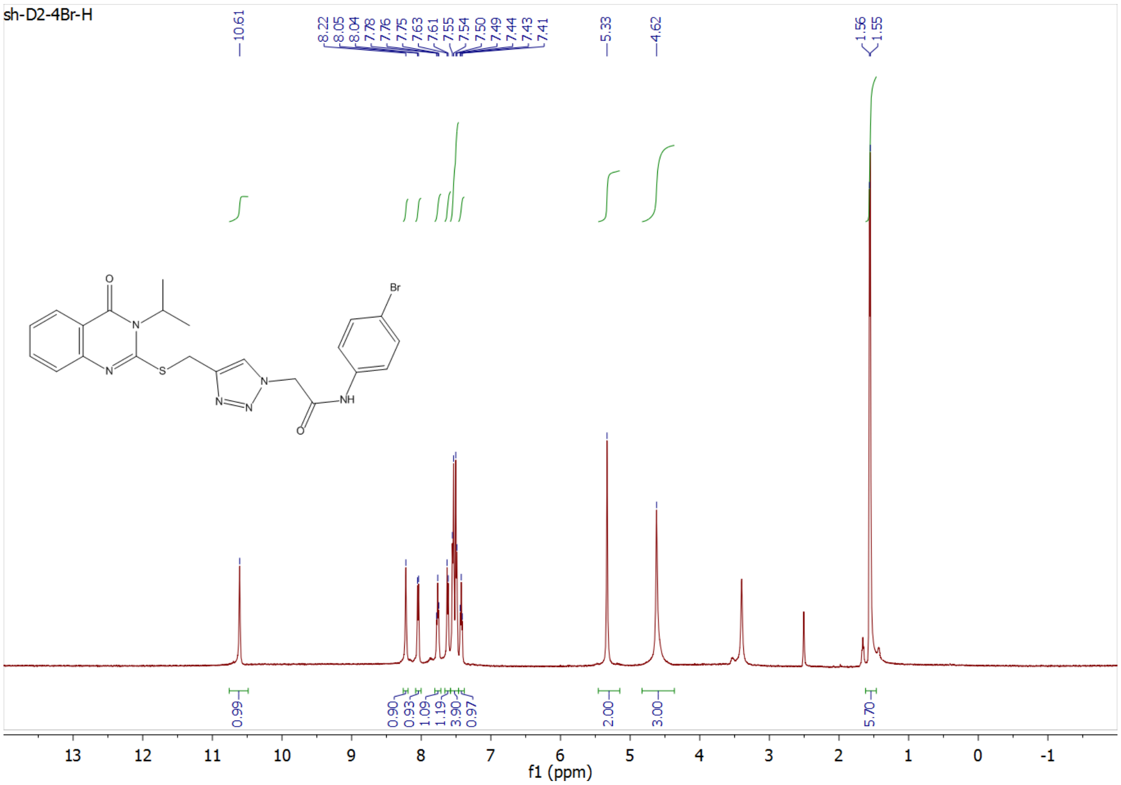

Figure S 5. ^{1}H NMR spectrum of N-(4-bromophenyl)-2-(4-(((3-isopropyl-4-oxo-3,4-dihydroquinazolin-2-yl)thio)methyl)-1H-1,2,3-triazol-1-yl)acetamide

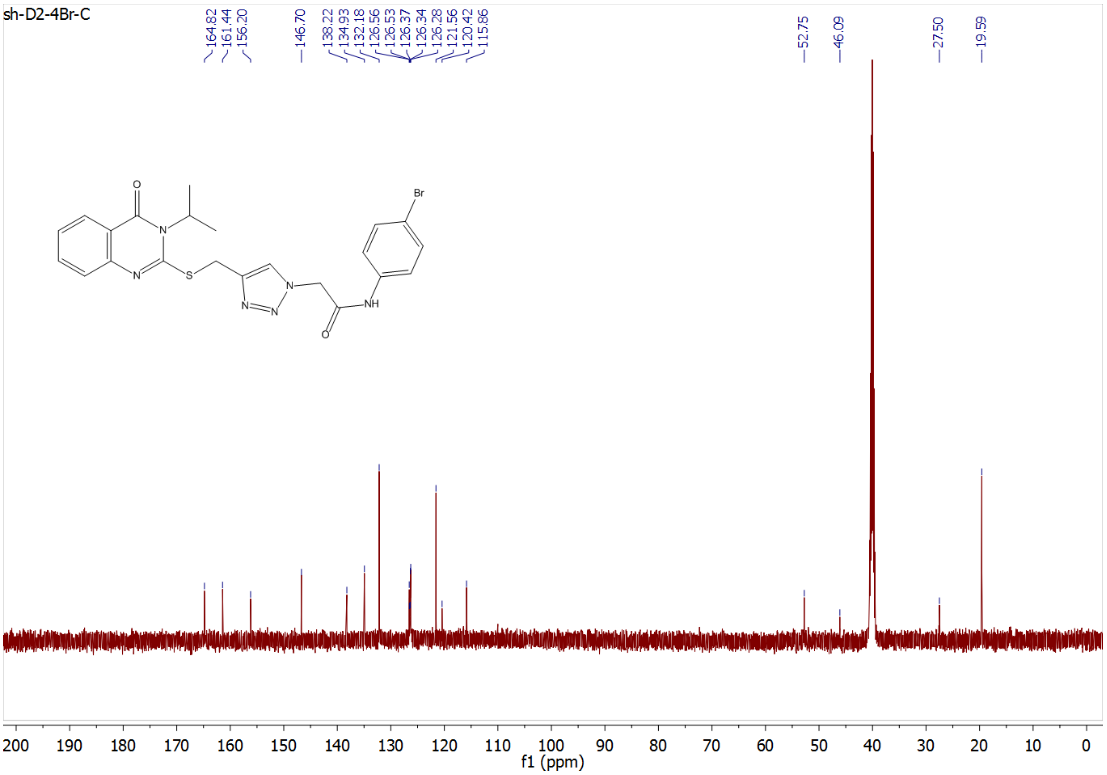

Figure S 6. ^{13}C NMR spectrum of N-(4-bromophenyl)-2-(4-(((3-isopropyl-4-oxo-3,4-dihydroquinazolin-2-yl)thio)methyl)-1H-1,2,3-triazol-1-yl)acetamide

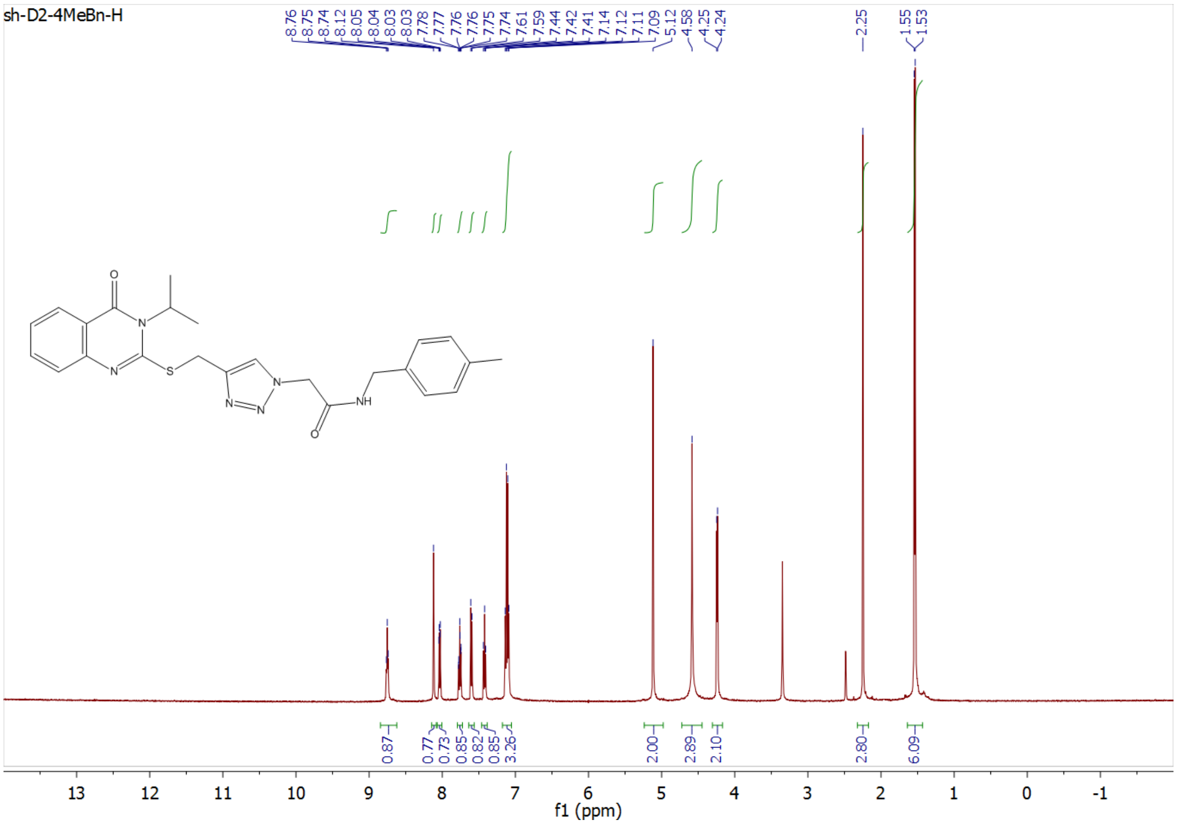

Figure S 7. ¹H NMR spectrum of 2-(4-(((3-isopropyl-4-oxo-3,4-dihydroquinazolin-2-yl)thio)methyl)-1H-1,2,3-triazol-1-yl)-N-(4-methylbenzyl)acetamide

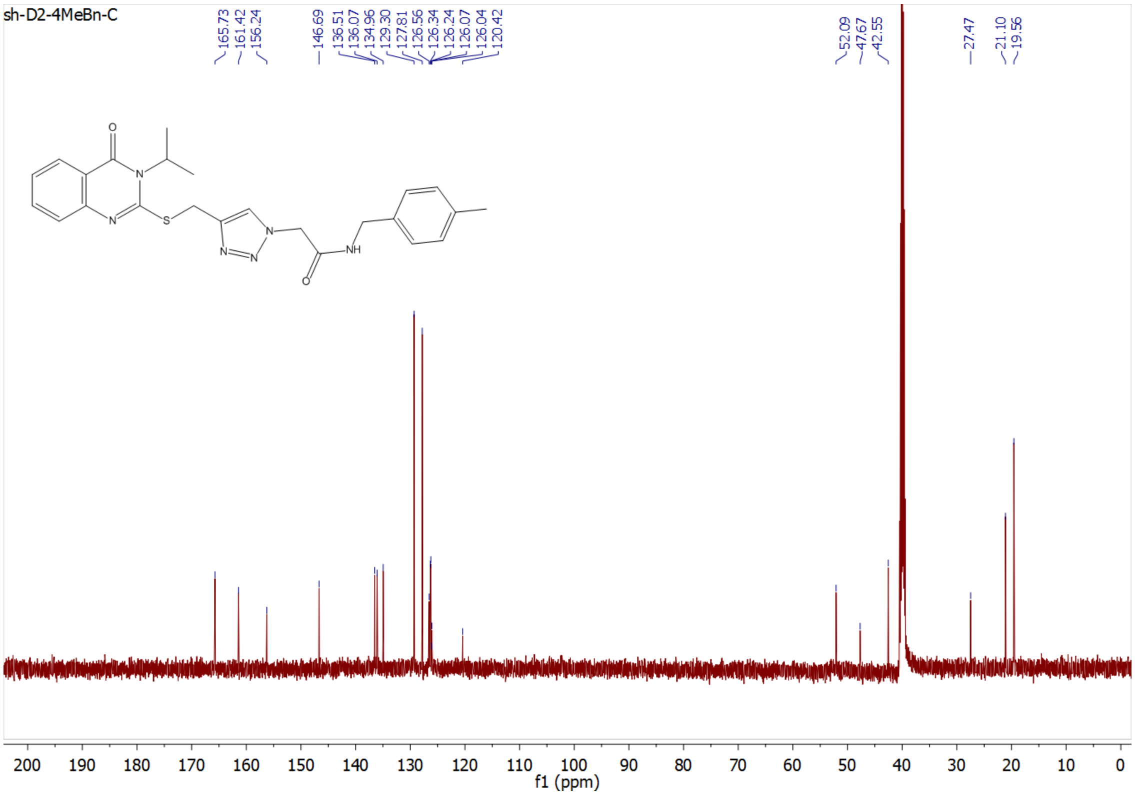

Figure S 8. ^{13}*C NMR spectrum of 2-(4-(((3-isopropyl-4-oxo-3,4-dihydroquinazolin-2-yl)thio)methyl)-1H-1,2,3-triazol-1-yl)-N-(4-methylbenzyl)acetamide*

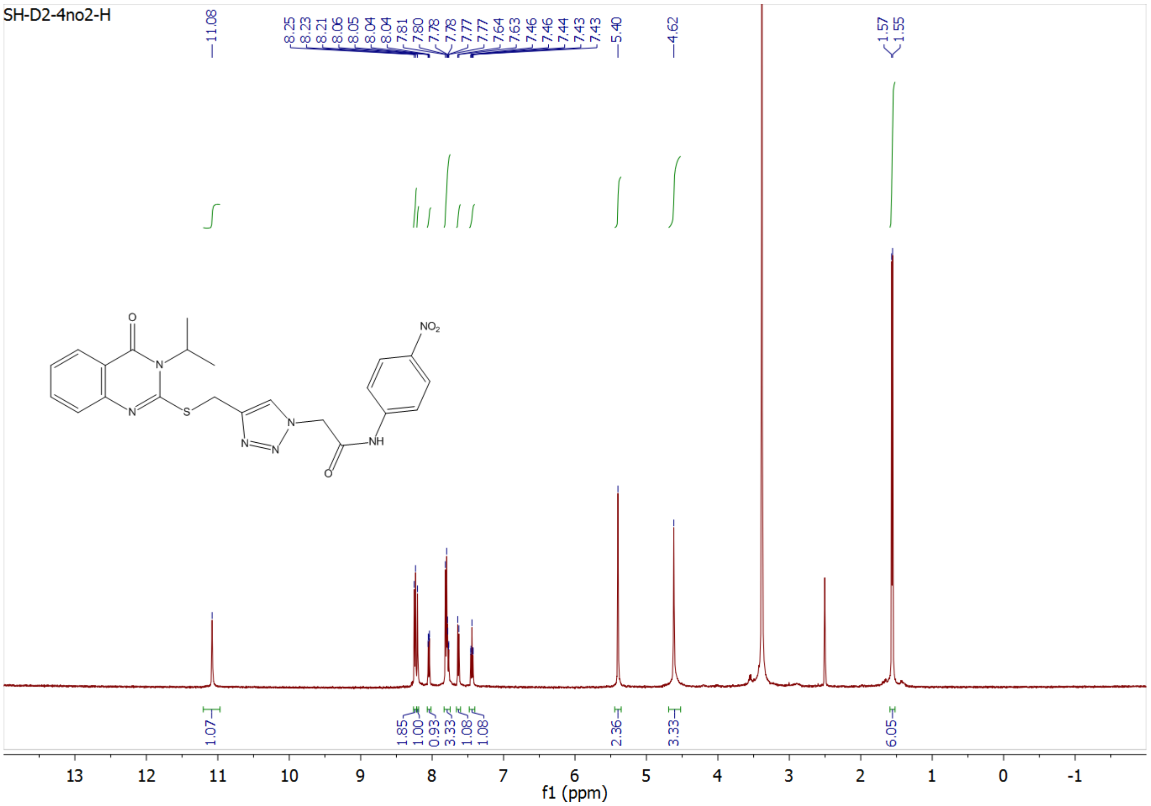

Figure S 9. ^{1}H NMR spectrum of 2-(4-(((3-isopropyl-4-oxo-3,4-dihydroquinazolin-2-yl)thio)methyl)-1H-1,2,3-triazol-1-yl)-N-(4-nitrophenyl)Acetamide

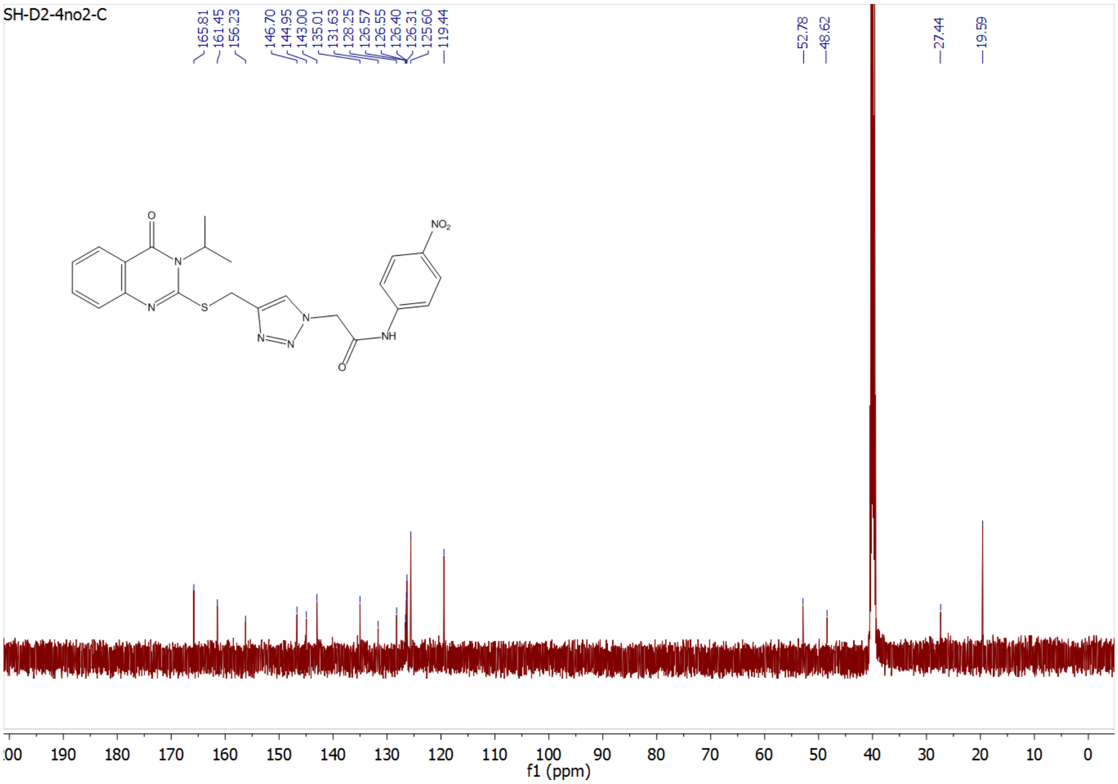

Figure S 10. 13C NMR spectrum of 2-(4-(((3-isopropyl-4-oxo-3,4-dihydroquinazolin-2-yl)thio)methyl)-1H-1,2,3-triazol-1-yl)-N-(4-nitrophenyl)Acetamide

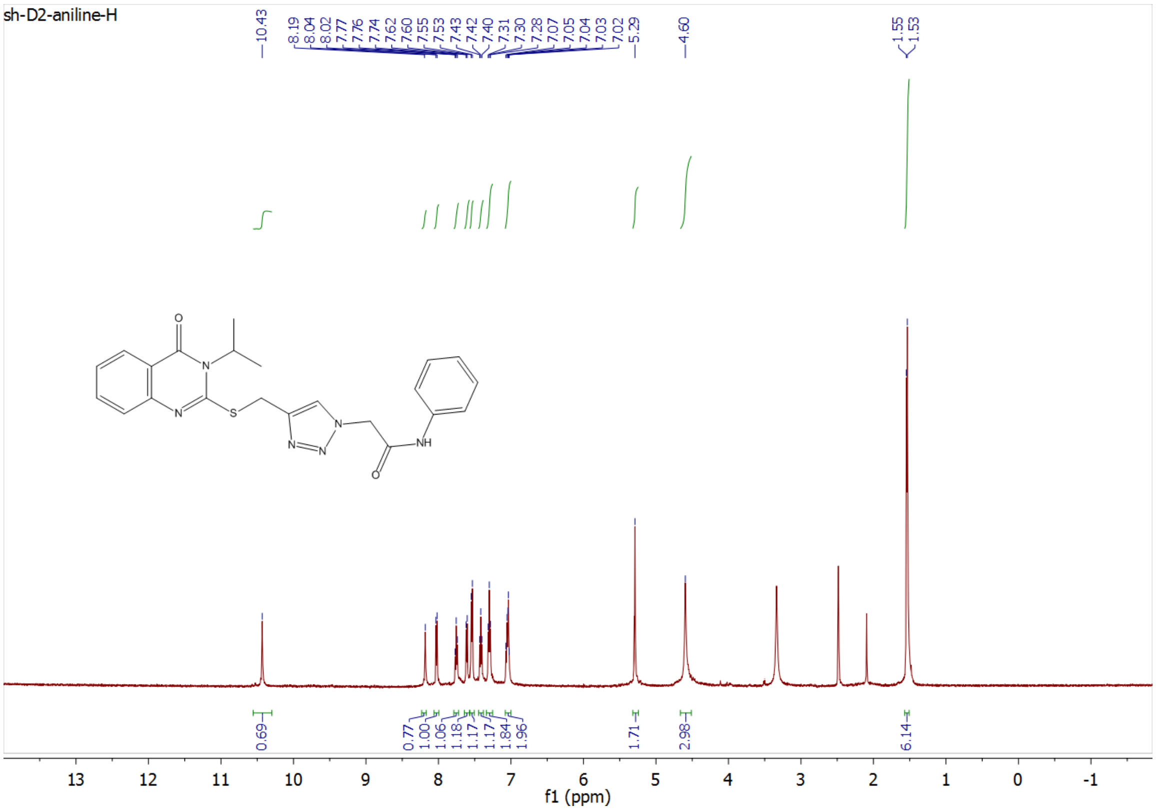

Figure S 11. ¹H NMR spectrum of 2-(4-(((3-isopropyl-4-oxo-3,4-dihydroquinazolin-2-yl)thio)methyl)-1H-1,2,3-triazol-1-yl)-N-phenylacetamide

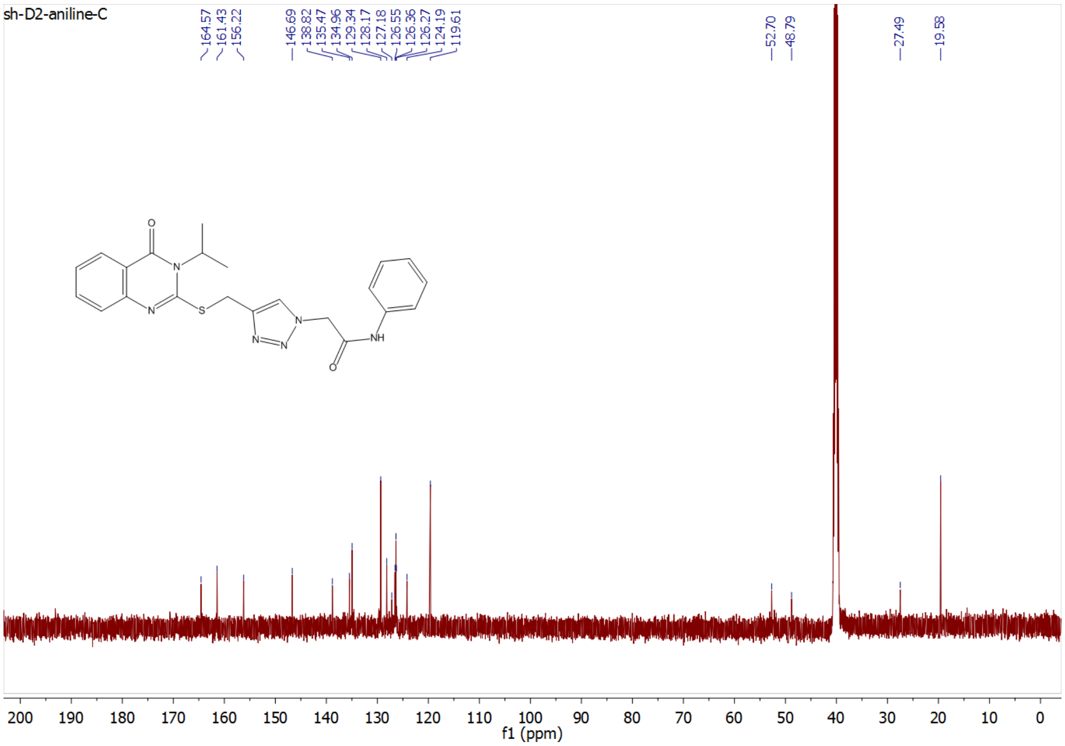

Figure S 12. ^{13}C NMR spectrum of 2-(4-(((3-isopropyl-4-oxo-3,4-dihydroquinazolin-2-yl)thio)methyl)-1H-1,2,3-triazol-1-yl)-N-phenylacetamide

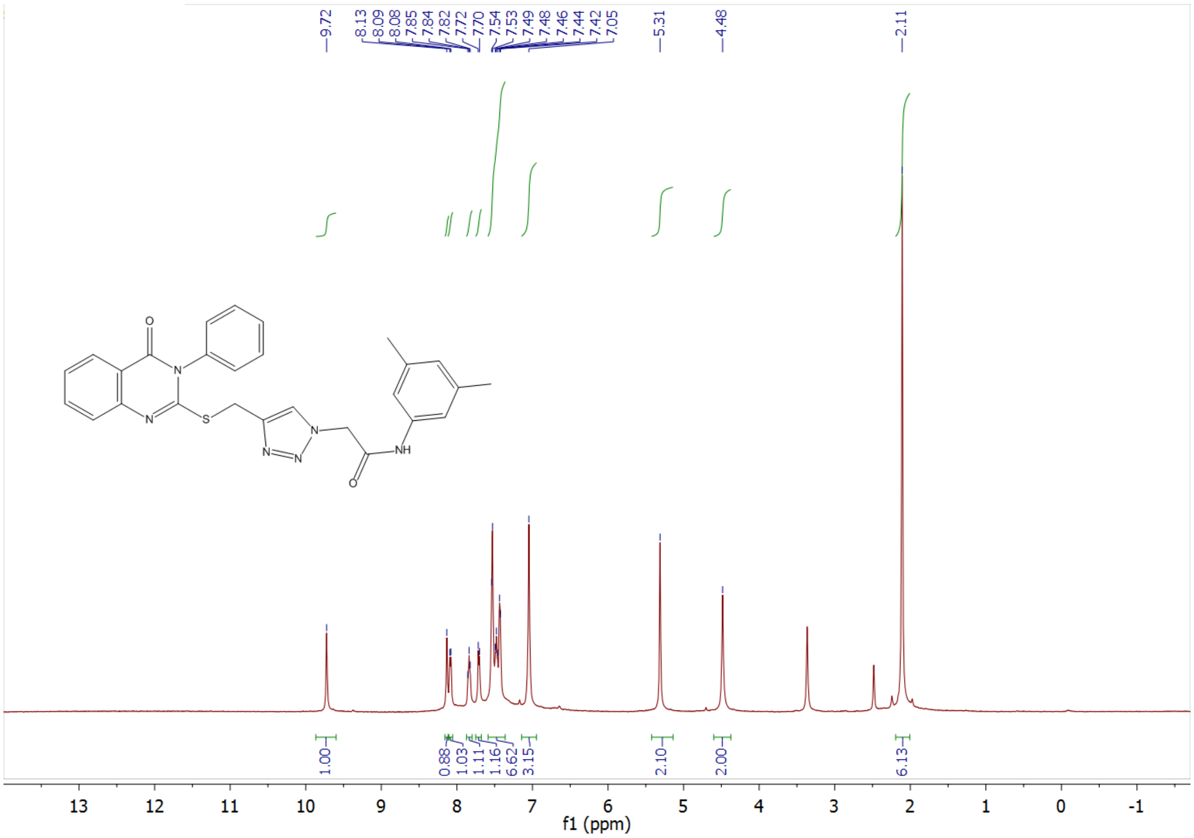

Figure S 13. ^{1}H spectrum of N-(3,5-dimethylphenyl)-2-(4-(((4-oxo-3-phenyl-3,4-dihydroquinazolin-2-yl)thio)methyl)-1H-1,2,3-triazol-1-yl)Acetamide

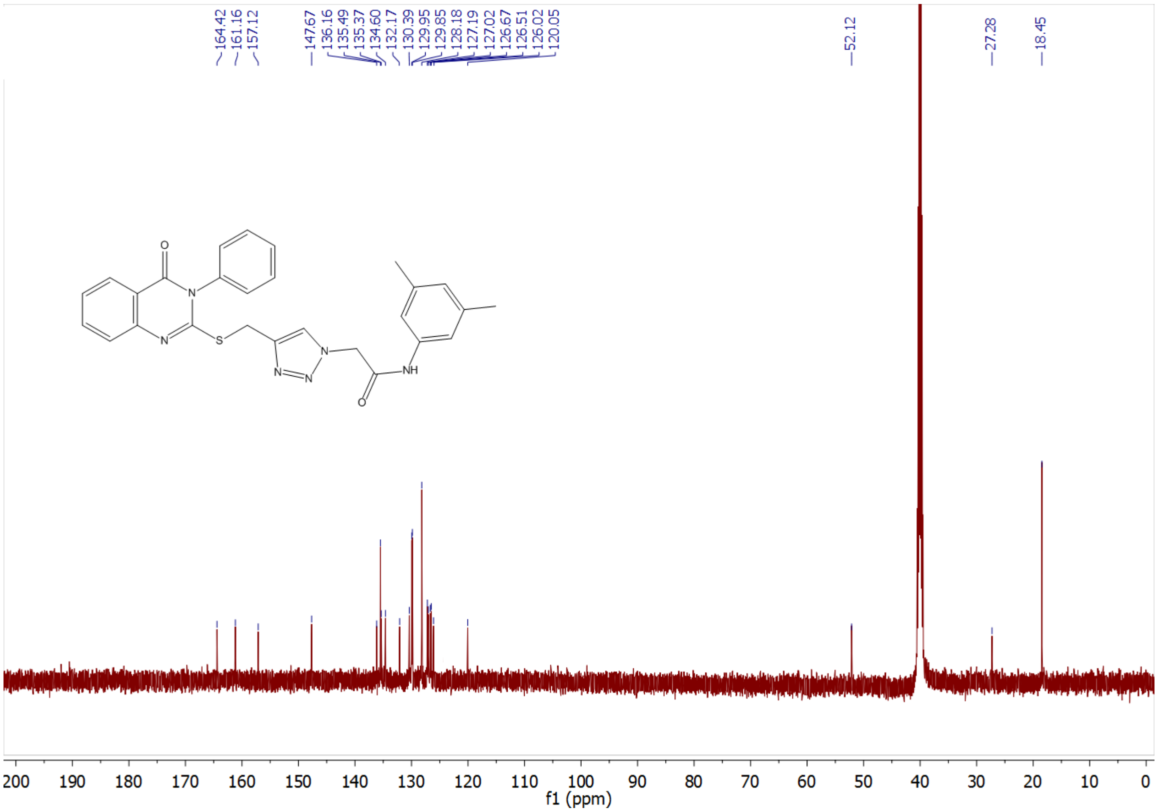

Figure S 14. ^{13}*C NMR spectrum of N-(3,5-dimethylphenyl)-2-(4-(((4-oxo-3-phenyl-3,4-dihydroquinazolin-2-yl)thio)methyl)-1H-1,2,3-triazol-1-yl)Acetamide*

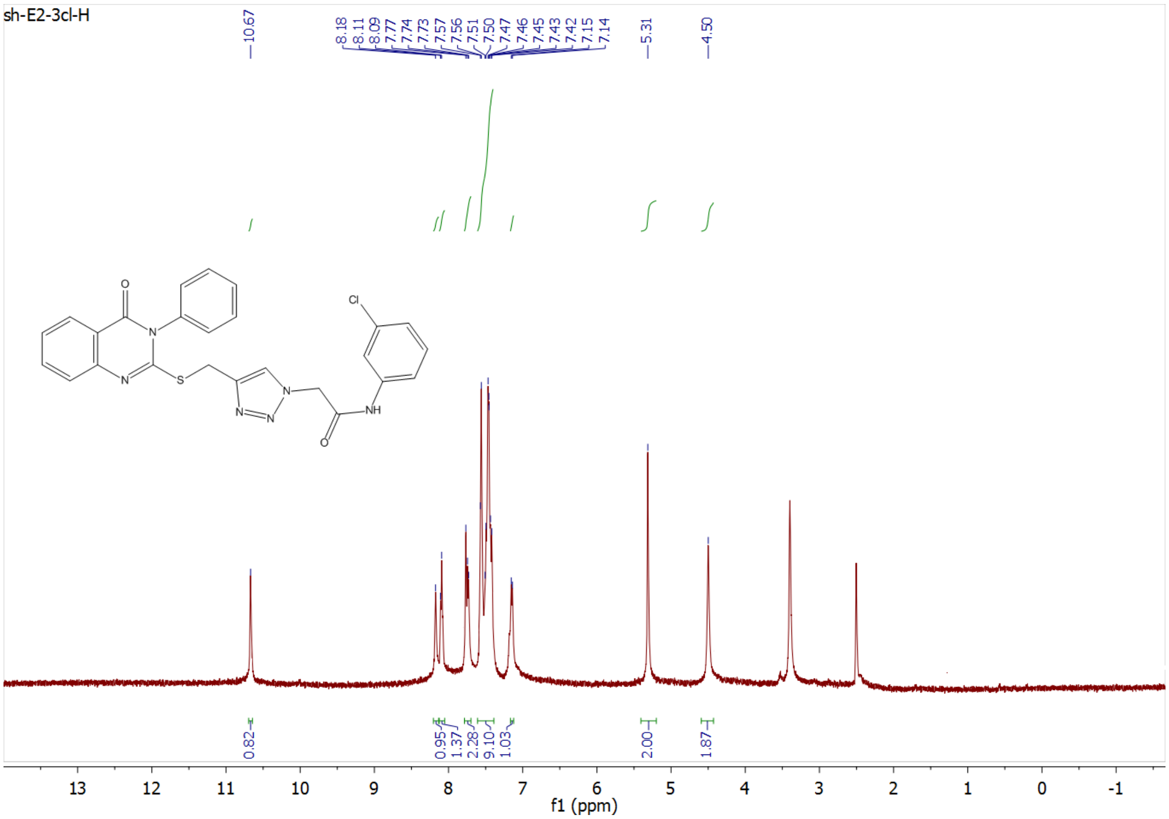

Figure S 15. ¹H NMR spectrum of N-(3-chlorophenyl)-2-(4-(((4-oxo-3-phenyl-3,4-dihydroquinazolin-2-yl)thio)methyl)-1H-1,2,3-triazol-1-yl)acetamide

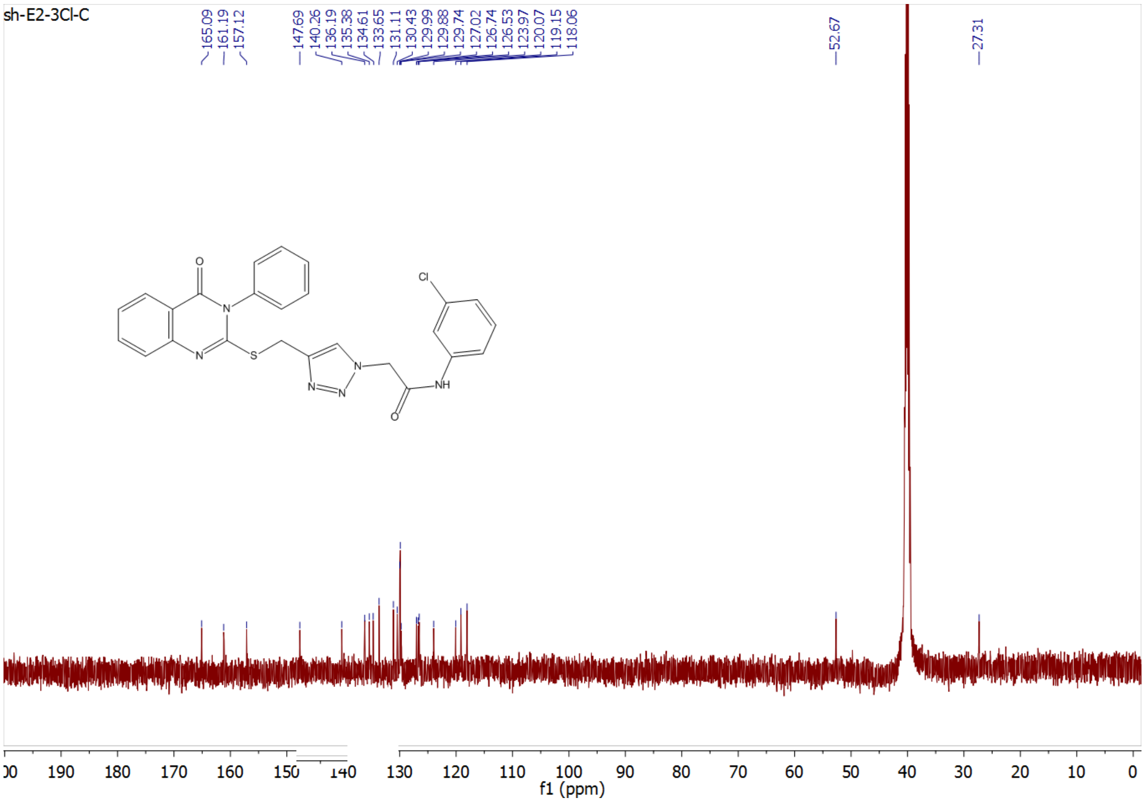

Figure S 16. ^{13}C NMR spectrum of N-(3-chlorophenyl)-2-(4-(((4-oxo-3-phenyl-3,4-dihydroquinazolin-2-yl)thio)methyl)-1H-1,2,3-triazol-1-yl)acetamide

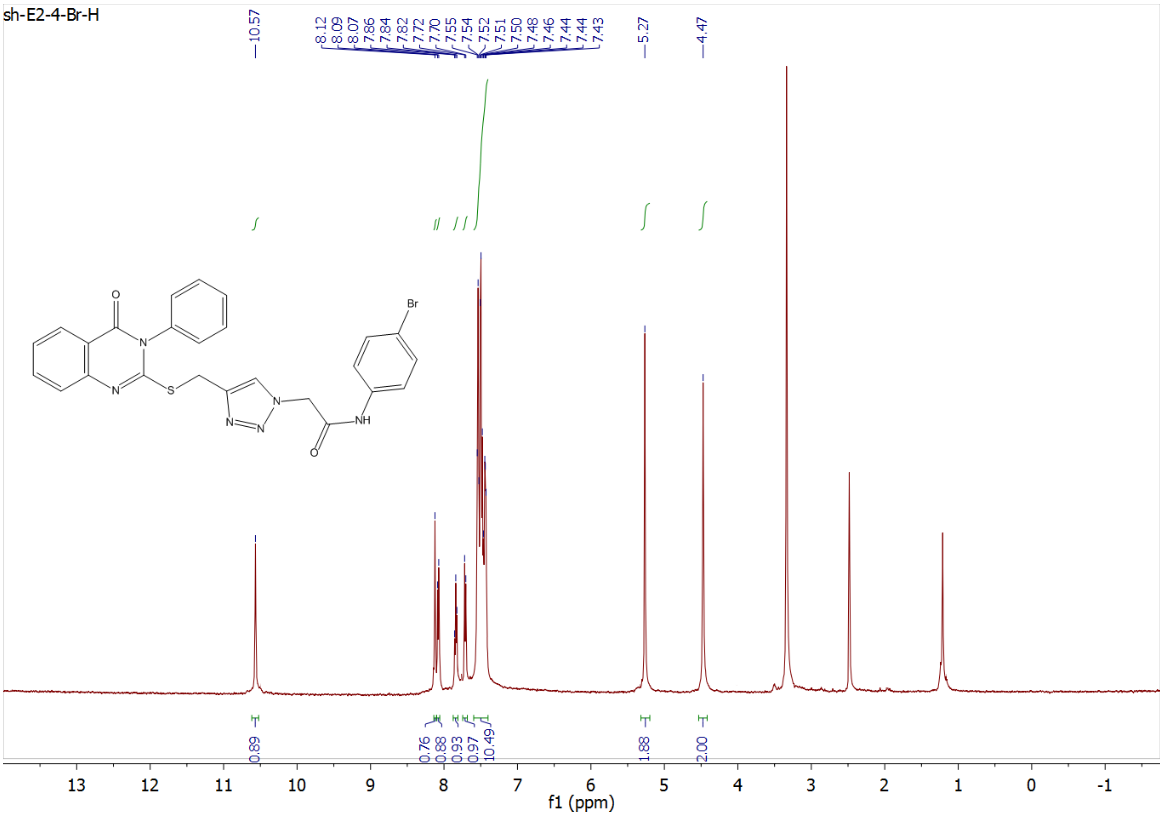

Figure S 17. ¹H NMR spectrum of N-(4-bromophenyl)-2-(4-(((4-oxo-3-phenyl-3,4-dihydroquinazolin-2-yl)thio)methyl)-1H-1,2,3-triazol-1-yl)acetamide

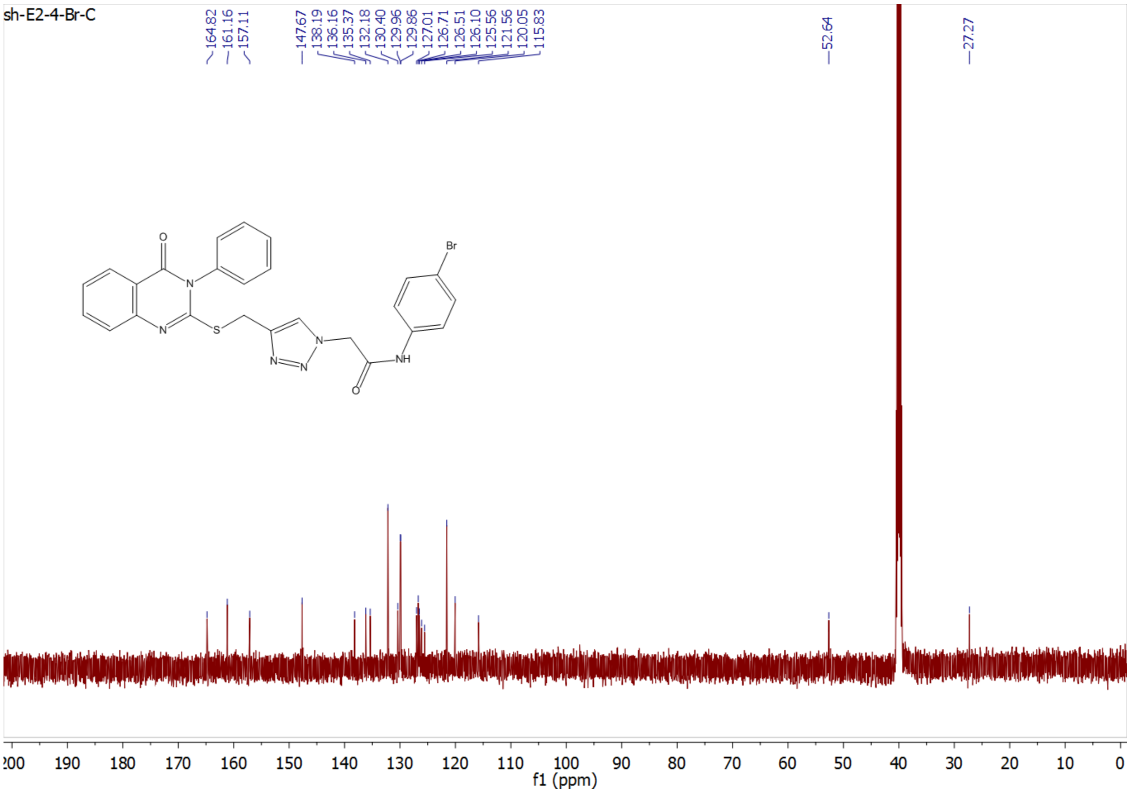

Figure S 18. ^{13}C NMR spectrum of N-(4-bromophenyl)-2-(4-(((4-oxo-3-phenyl-3,4-dihydroquinazolin-2-yl)thio)methyl)-1H-1,2,3-triazol-1-yl)acetamide

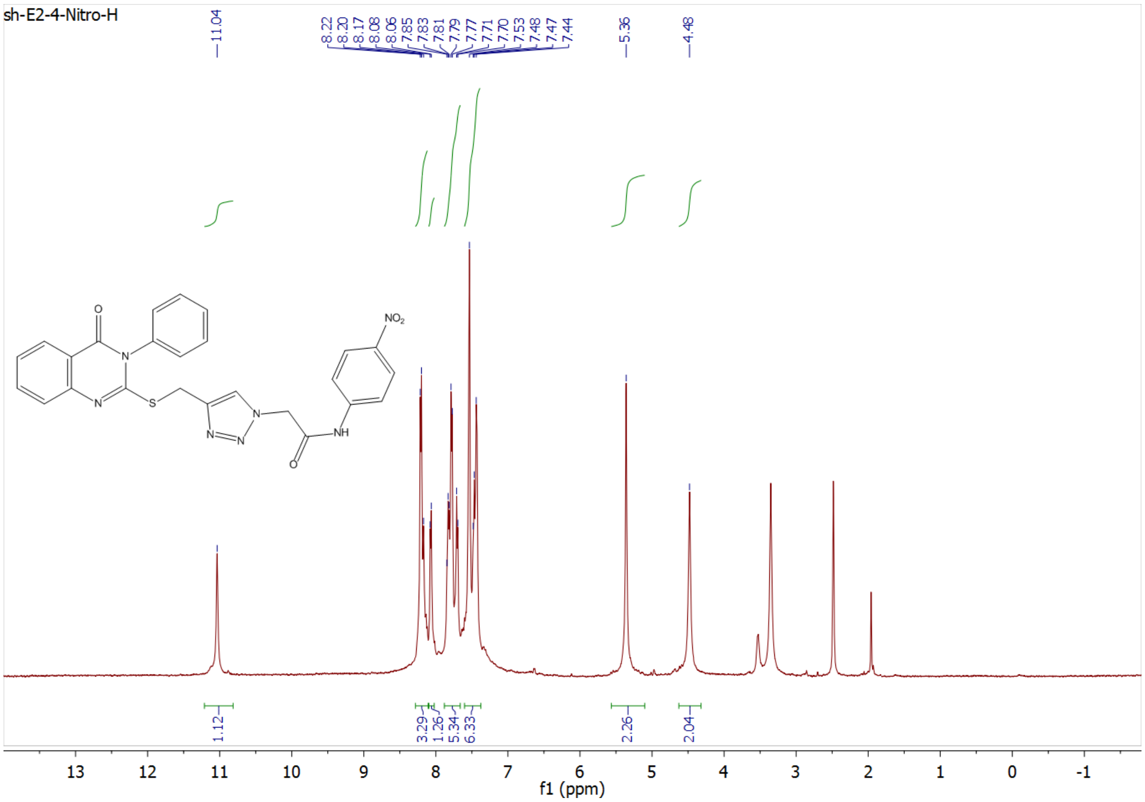

Figure S 19. ^{1}H NMR spectrum of N-(4-nitrophenyl)-2-(4-(((4-oxo-3-phenyl-3,4-dihydroquinazolin-2-yl)thio)methyl)-1H-1,2,3-triazol-1-yl)acetamide

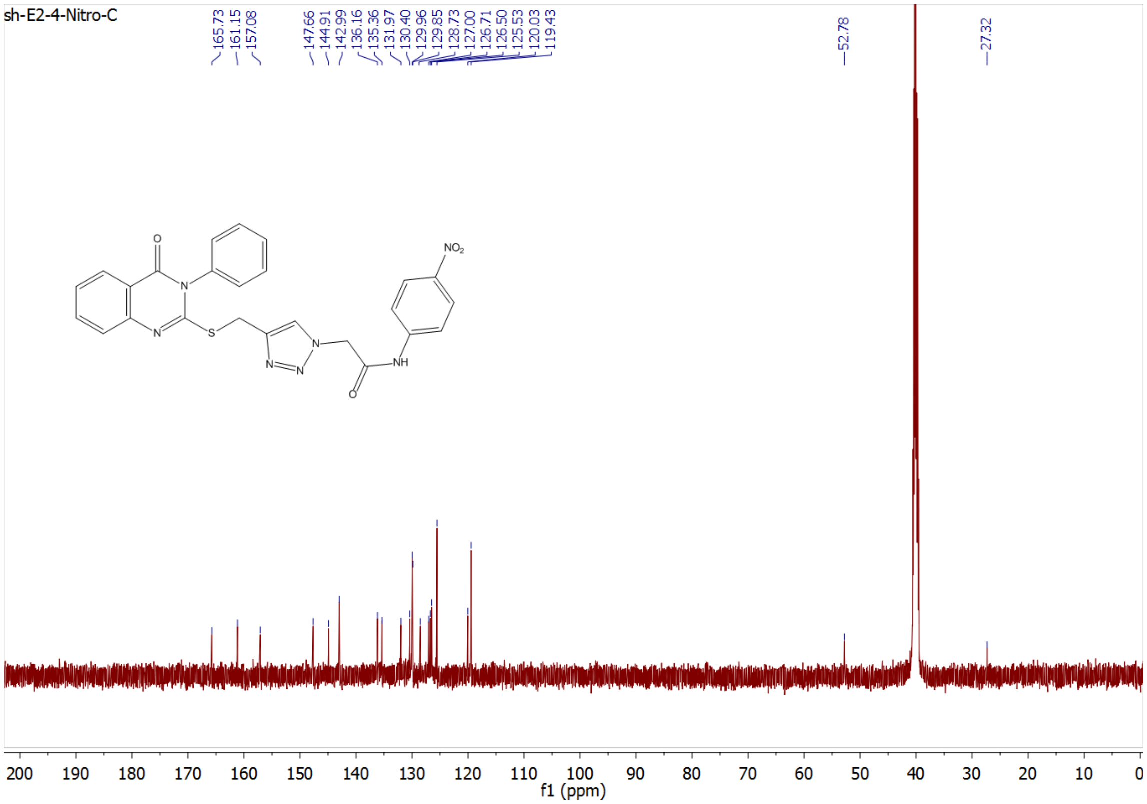

Figure S 20. ^{13}C NMR spectrum of N-(4-nitrophenyl)-2-(4-(((4-oxo-3-phenyl-3,4-dihydroquinazolin-2-yl)thio)methyl)-1H-1,2,3-triazol-1-yl)acetamide

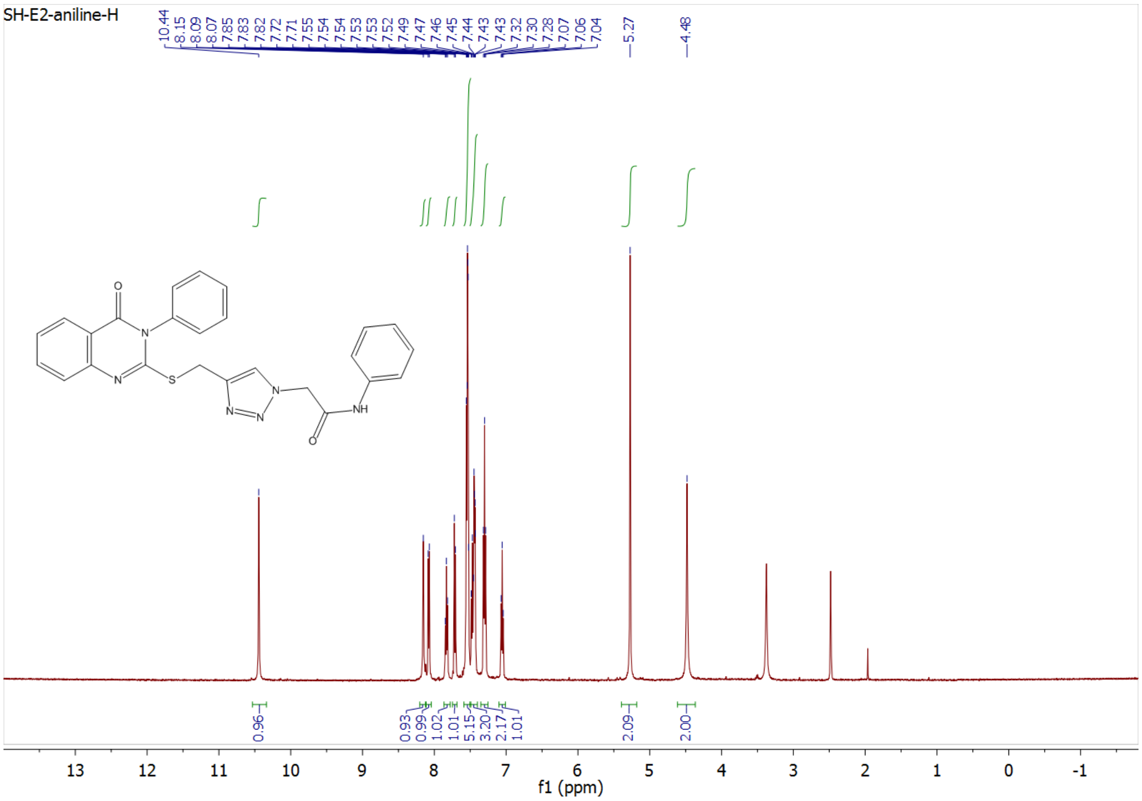

Figure S 21. ^{1}H NMR spectrum of 2-(4-(((4-oxo-3-phenyl-3,4-dihydroquinazolin-2-yl)thio)methyl)-1H-1,2,3-triazol-1-yl)-N-phenylacetamide

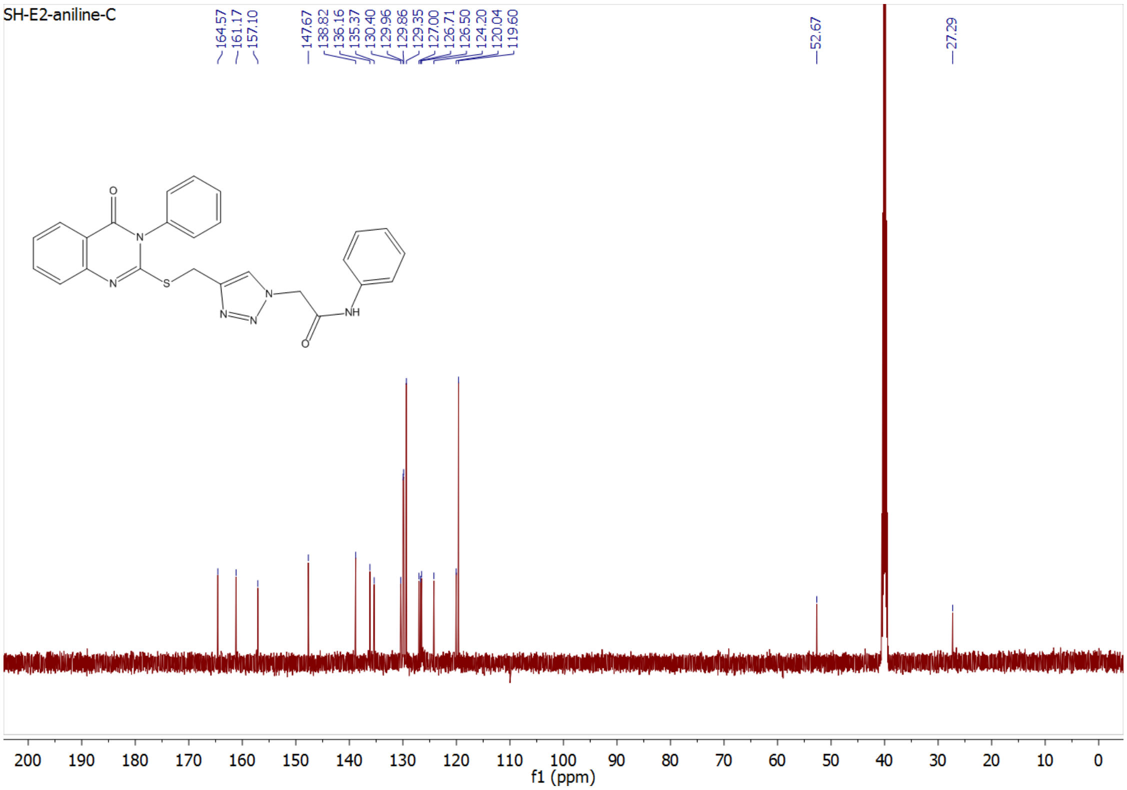

Figure S 22. ^{13}C NMR spectrum of 2-(4-(((4-oxo-3-phenyl-3,4-dihydroquinazolin-2-yl)thio)methyl)-1H-1,2,3-triazol-1-yl)-N-phenylacetamide

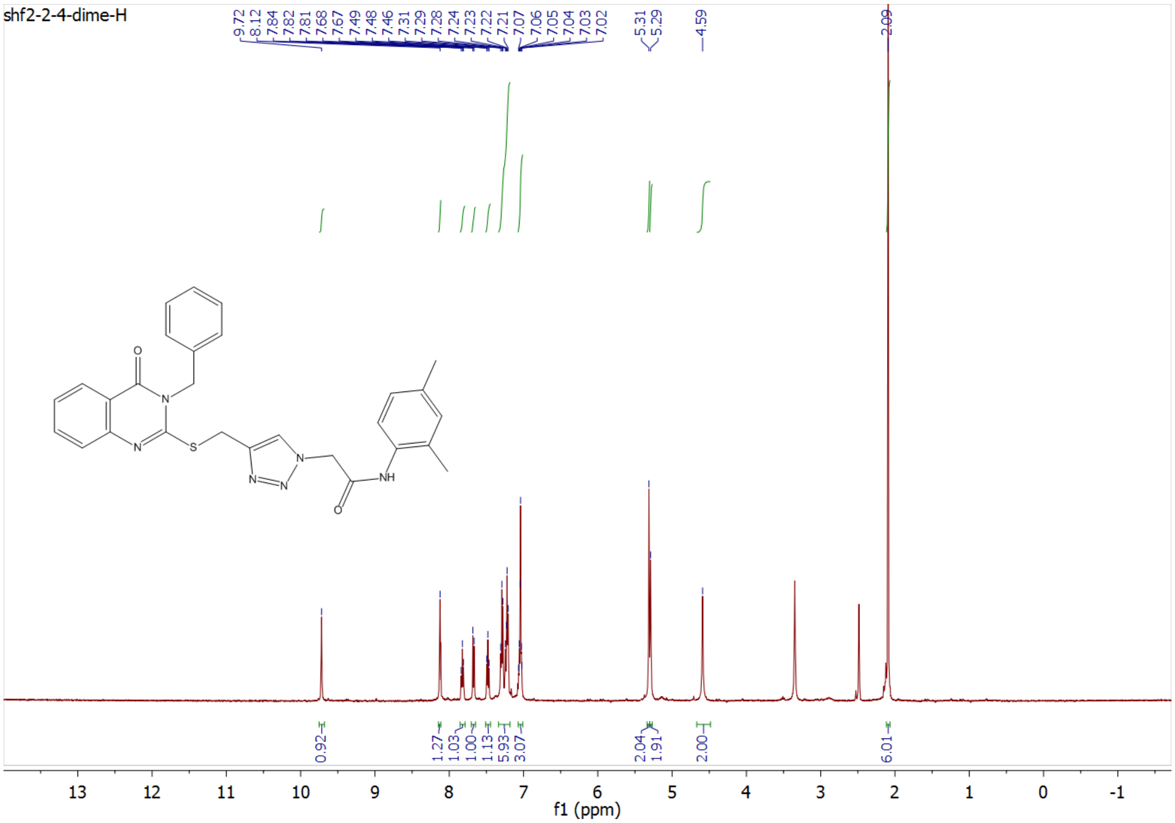

Figure S 23. ^{1}H NMR spectrum of 2-(4-(((3-benzyl-4-oxo-3,4-dihydroquinazolin-2-yl)thio)methyl)-1H-1,2,3-triazol-1-yl)-N-(2,4-dimethylphenyl)acetamide

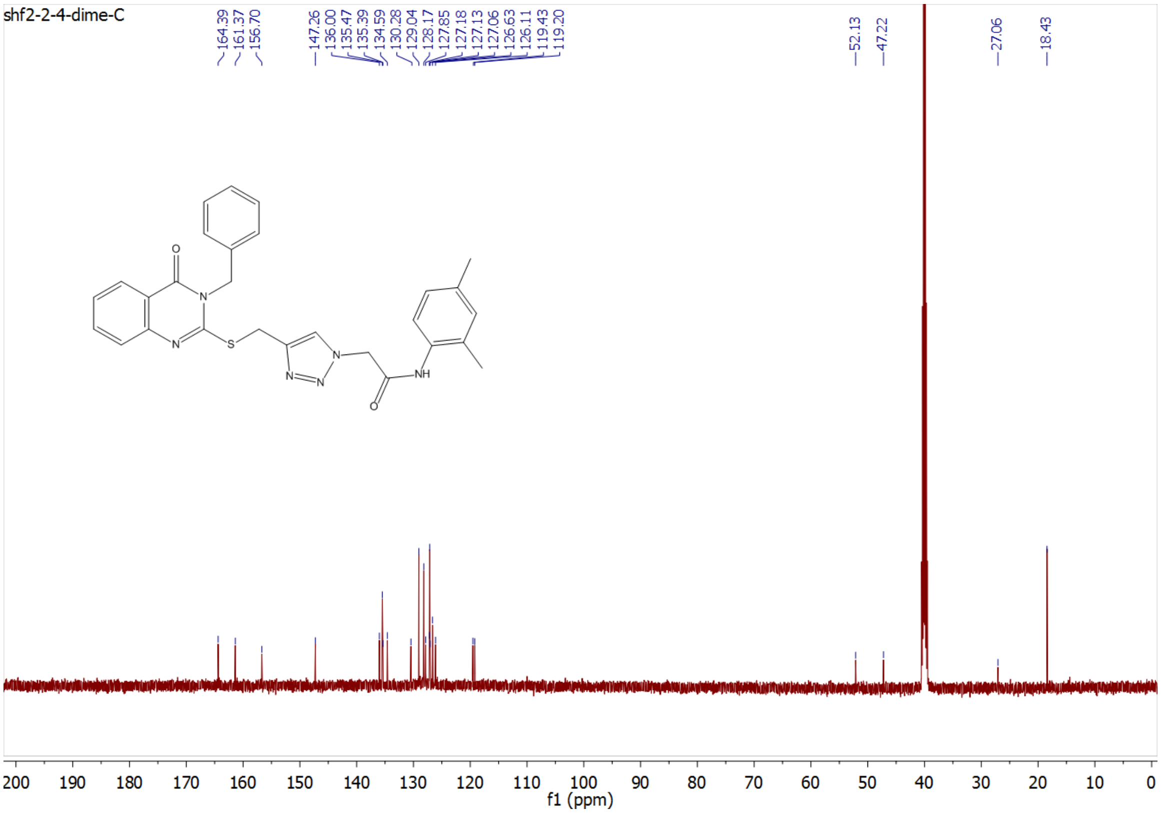

Figure S 24. ^{13}C NMR spectrum of 2-(4-(((3-benzyl-4-oxo-3,4-dihydroquinazolin-2-yl)thio)methyl)-1H-1,2,3-triazol-1-yl)-N-(2,4-dimethylphenyl)acetamide

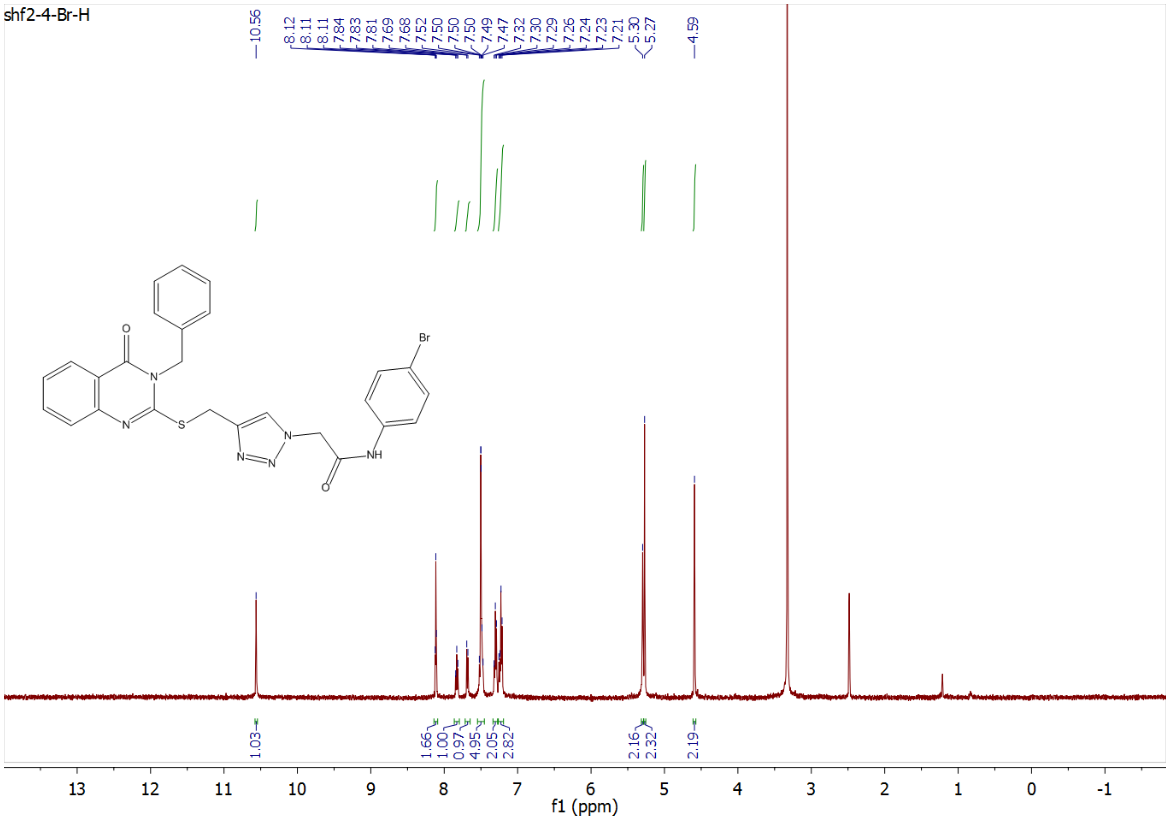

Figure S 25. ^{1}H NMR spectrum of 2-(4-(((3-benzyl-4-oxo-3,4-dihydroquinazolin-2-yl)thio)methyl)-1H-1,2,3-triazol-1-yl)-N-(4-bromophenyl)acetamide

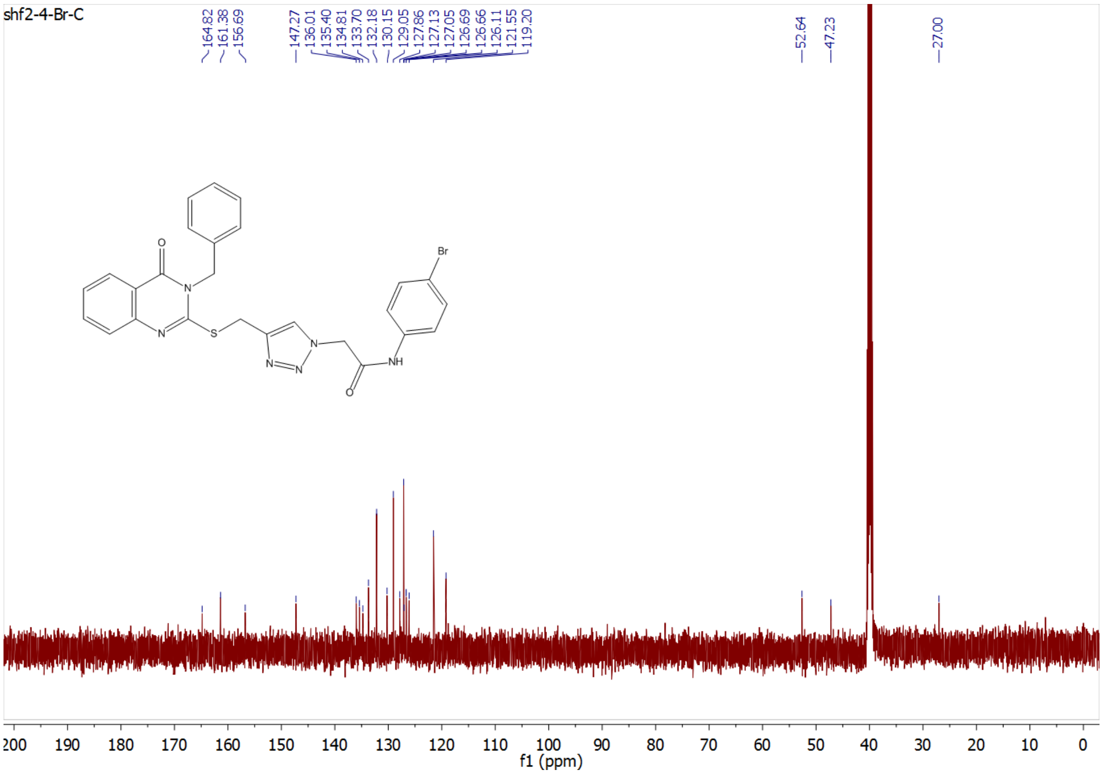

Figure S 26. ^{13}C NMR spectrum of 2-(4-(((3-benzyl-4-oxo-3,4-dihydroquinazolin-2-yl)thio)methyl)-1H-1,2,3-triazol-1-yl)-N-(4-bromophenyl)acetamide

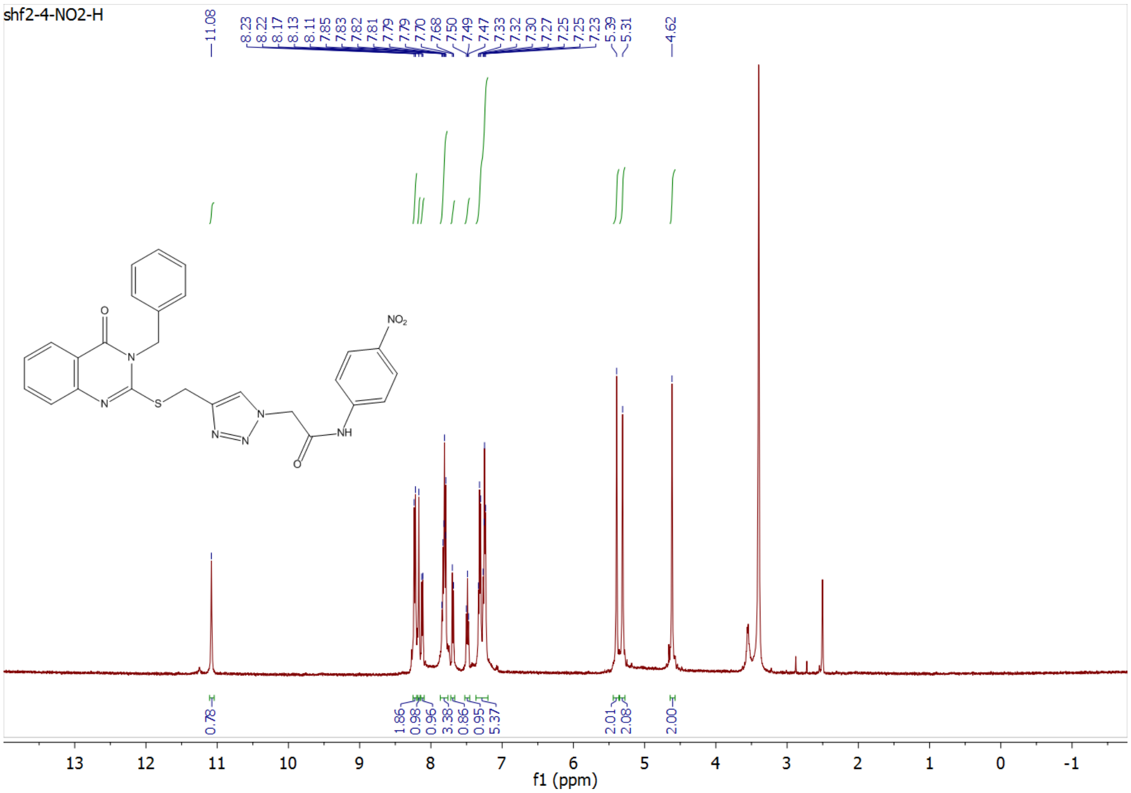

Figure S 27. ^{1}H NMR spectrum of 2-(4-(((3-benzyl-4-oxo-3,4-dihydroquinazolin-2-yl)thio)methyl)-1H-1,2,3-triazol-1-yl)-N-(4-nitrophenyl)acetamide

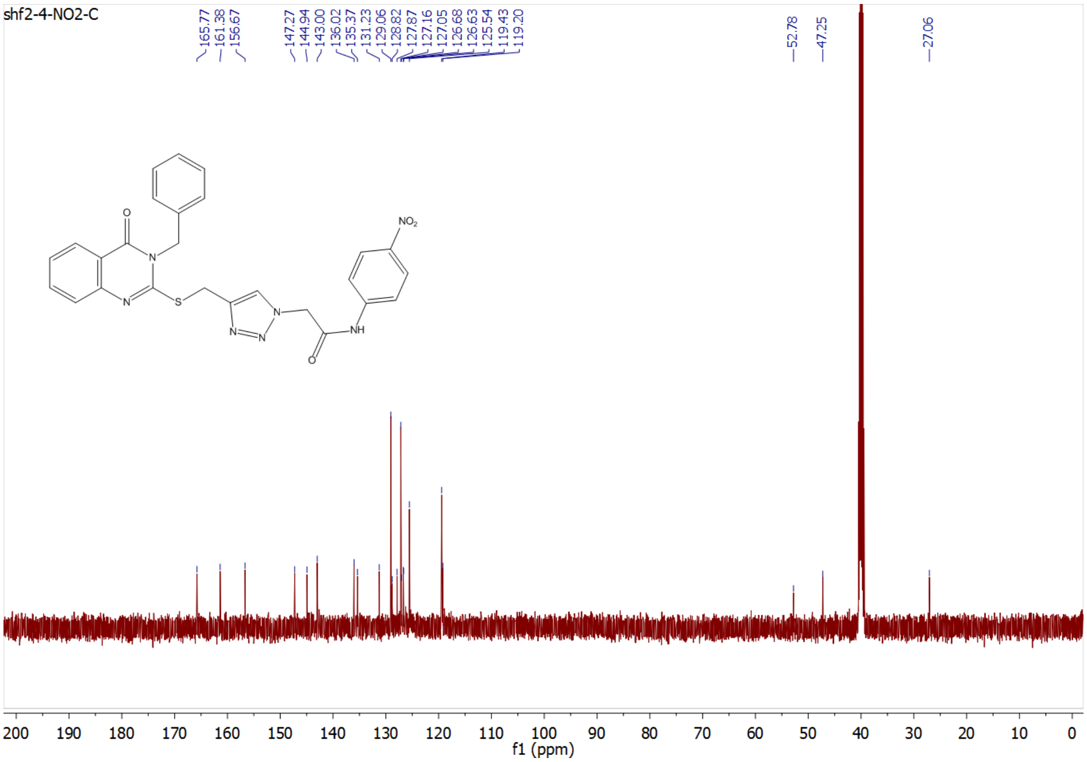

Figure S 28. ^{13}C NMR spectrum of 2-(4-(((3-benzyl-4-oxo-3,4-dihydroquinazolin-2-yl)thio)methyl)-1H-1,2,3-triazol-1-yl)-N-(4-nitrophenyl)acetamide

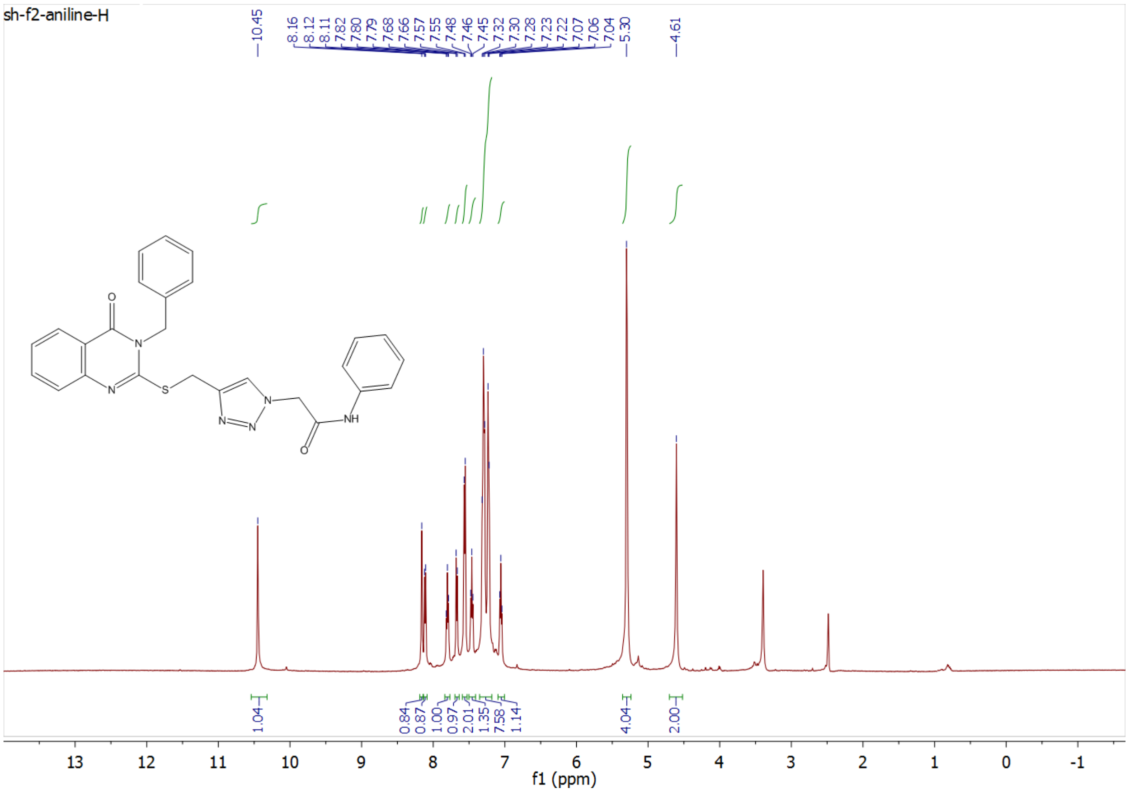

Figure S 29. ^{1}H NMR spectrum of 2-(4-(((3-benzyl-4-oxo-3,4-dihydroquinazolin-2-yl)thio)methyl)-1H-1,2,3-triazol-1-yl)-N-phenylacetamide

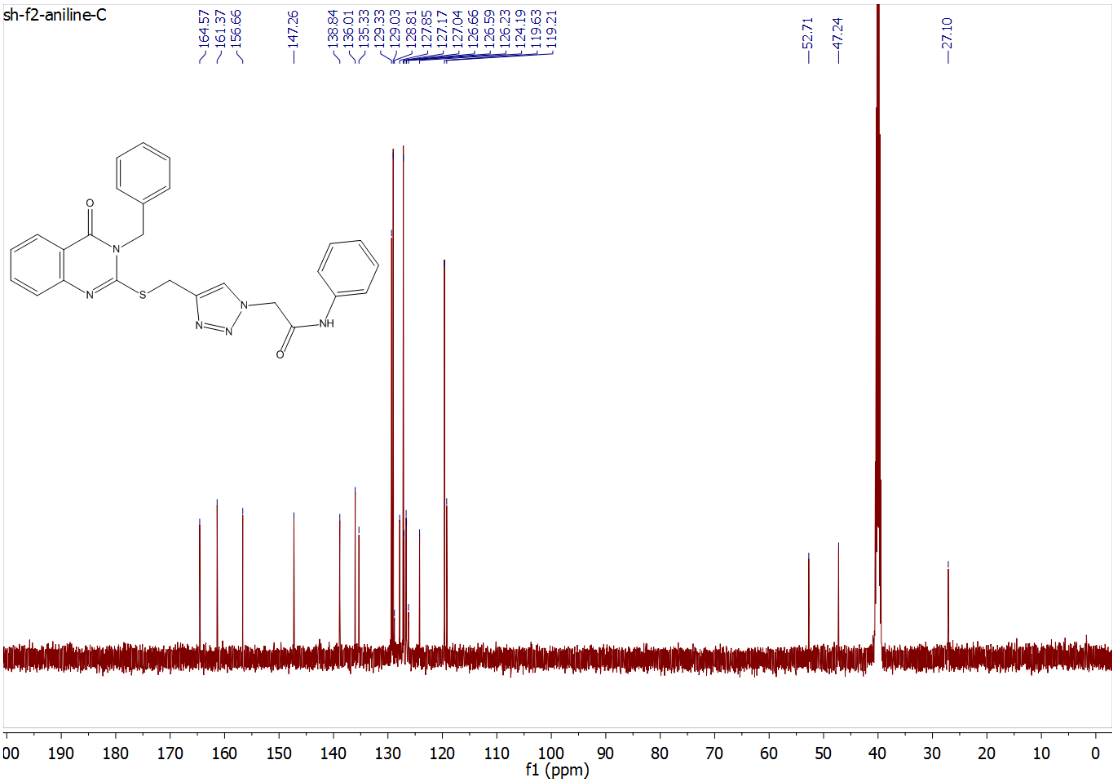

Figure S 30. 13C NMR spectrum of 2-(4-(((3-benzyl-4-oxo-3,4-dihydroquinazolin-2-yl)thio)methyl)-1H-1,2,3-triazol-1-yl)-N-phenylacetamide

Figure S 31. 1H NMR spectrum of 2-(4-(((3-benzyl-4-oxo-3,4-dihydroquinazolin-2-yl)thio)methyl)-1H-1,2,3-triazol-1-yl)-N-(3-chlorophenyl)acetamide

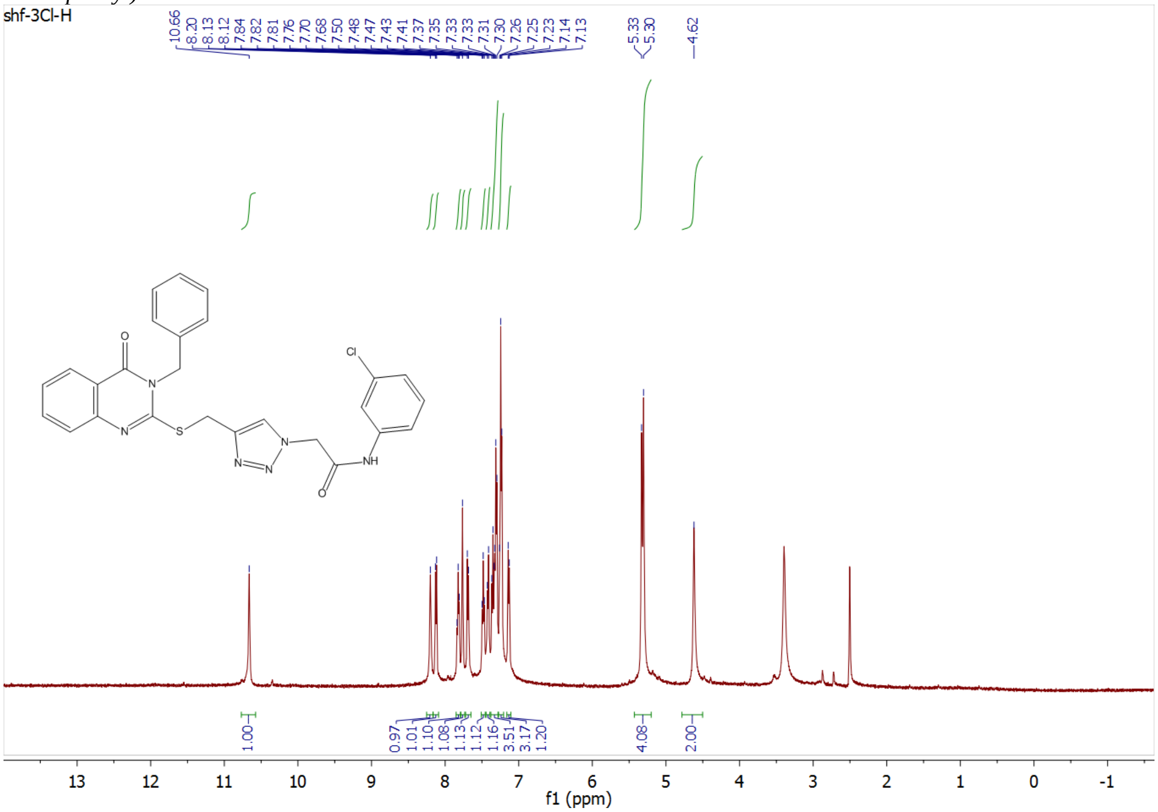

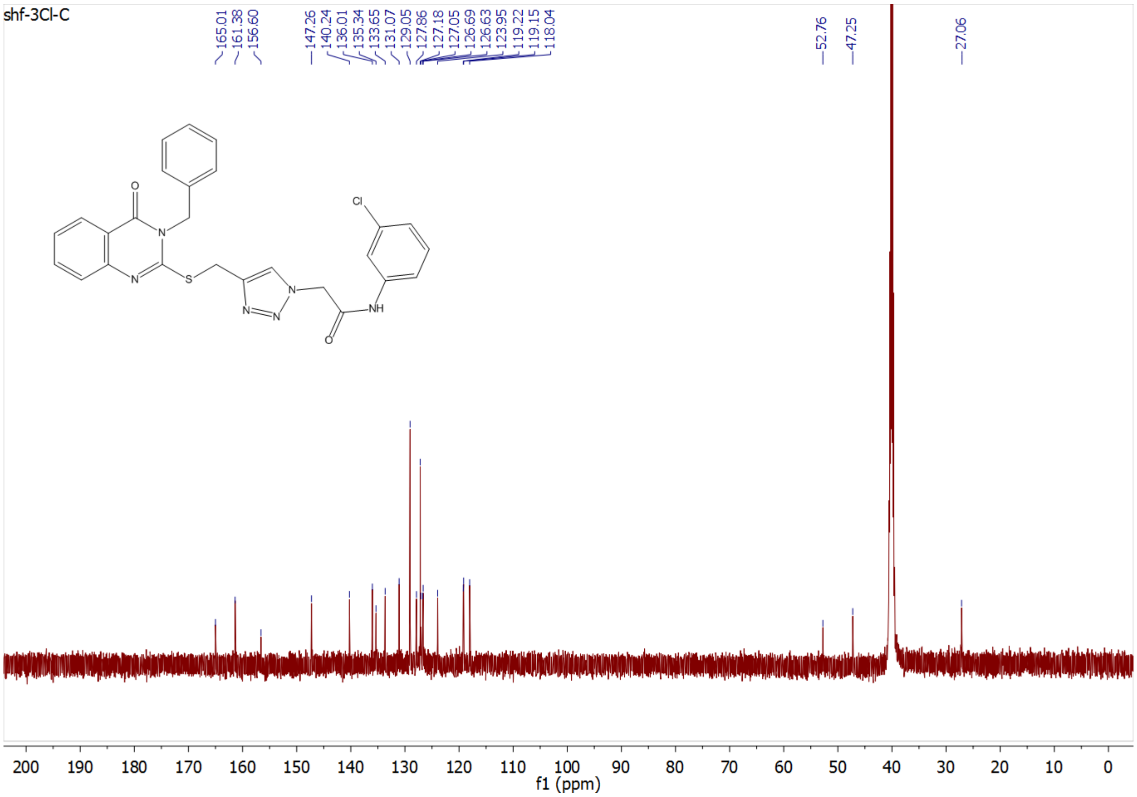

Figure S 32. ^{13}C NMR spectrum of 2-(4-(((3-benzyl-4-oxo-3,4-dihydroquinazolin-2-yl)thio)methyl)-1H-1,2,3-triazol-1-yl)-N-(3-chlorophenyl)acetamide
